# Facile solvent-free modified biochar for removal of mixed steroid hormones and heavy metals: isotherm and kinetic studies

**DOI:** 10.1186/s13065-023-01071-5

**Published:** 2023-11-20

**Authors:** Sefiu Olaitan Amusat, Temesgen Girma Kebede, Edward Ndumiso Nxumalo, Simiso Dube, Mathew Muzi Nindi

**Affiliations:** 1 Department of Chemistry, College of Science, Engineering, and Technology, University of South Africa, The Science Campus, Florida Park, Corner Christian de Wet & Pioneer Avenue, Florida, 1709 South Africa; 2 Institute for Nanotechnology and Water Sustainability (iNanoWS), College of Science, Engineering, and Technology, The Science Campus, University of South Africa, Corner Christian de Wet & Pioneer Avenue, Florida, 1709 South Africa

**Keywords:** Ball milled biochar, Heavy metals, Steroid hormones, Adsorption, Isotherm, Kinetics

## Abstract

Water contamination has become a global challenge to human survival. Non-biodegradable heavy metal cations and steroid hormones could accumulate in the human body and could result in serious health problems. In this study, we prepared biochar from waste shells of African star apples and modified biochar using a solvent-free ball milling facile method. The X-ray photoelectron spectrometer (XPS) and Fourier transform infrared spectroscopy (FTIR) analysis revealed biochar functional groups in C=C, C–O, and C=O. Brunauer Emmett Teller (BET) was used to determine the surface area, the surface area of ball-milled biochar obtained at 550 °C (BASA550) increased from 174 m^2^/g to 304 m^2^/g after modification. The Langmuir and Freundlich adsorption isotherms best described the experimental adsorption data with R_L_ < 1 and 1/*n* < 1 and a high degree of agreement of R^2^ data; Langmuir (R^2^ = 0.9291–0.9992) and Freundlich (R^2^ = 0.9077–0.9974). The adsorption kinetic studies using pseudo-first-order and pseudo-second-order models revealed that the pseudo-second-order model accurately described the adsorption process). The application of the BASA550 for treating wastewater samples showed a good percentage of removal. The removal percentage for cadmium, nickel, and lead was recorded as 92.96%, 90.89%, and 90.29%, respectively. The percentage removal in the influent and effluent were found to be 85.06%, 83.87%, 84.73%, and 89.37%, 86.48%, and 87.40%, respectively. The maximum percentage removal of steroid hormones from ultrapure water ranged from 84.20 to 89.63%, while from the spiked effluent and influent the percentage removal of 78.91–87.81% and 73.58–84.51% were obtained. The reusability of the ball-milled biochar was investigated and the result showed that the adsorbent (BASA550) had a good reusability potential for the first four cycles.

**Graphical Abstract:**

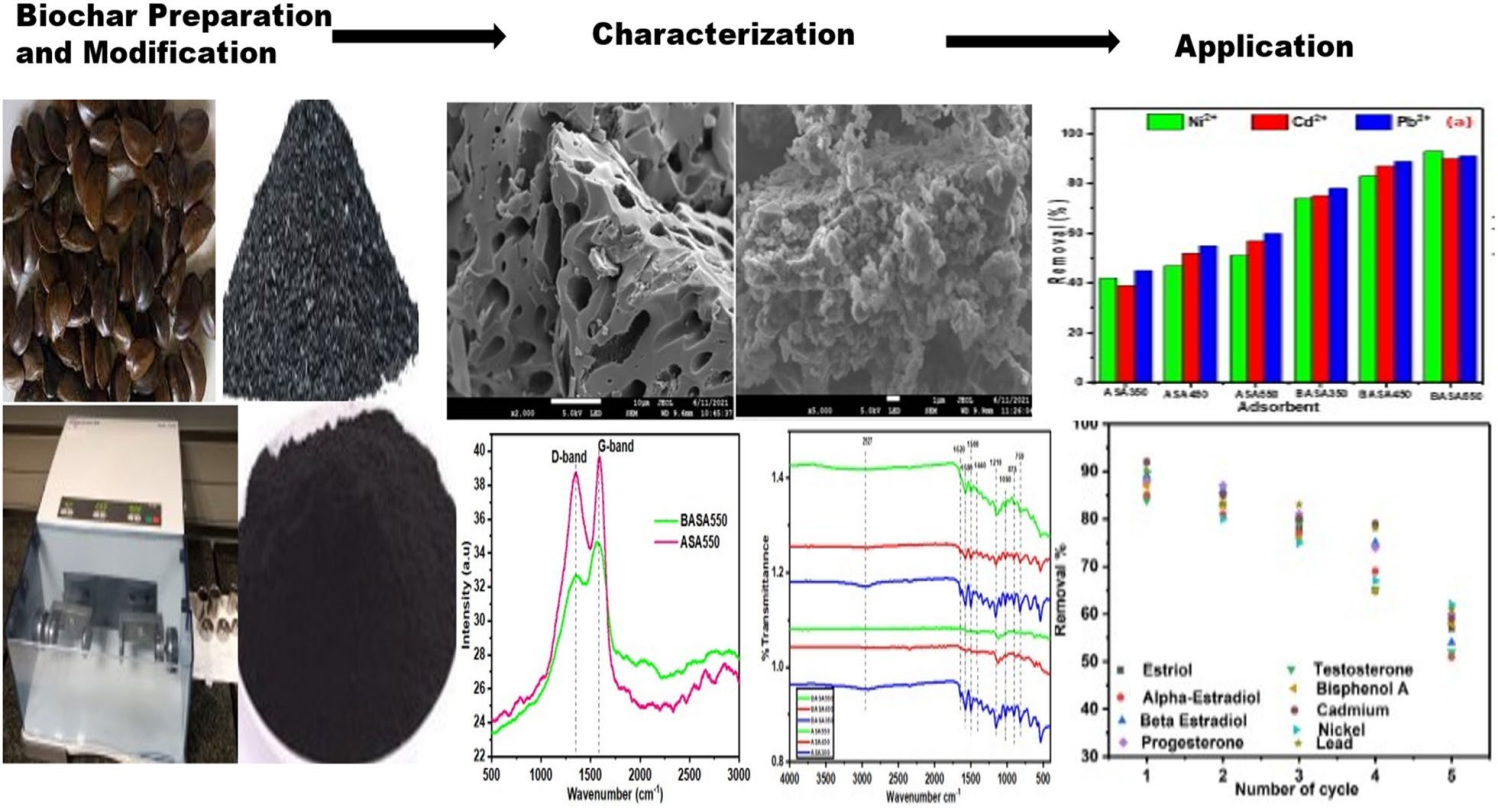

**Supplementary Information:**

The online version contains supplementary material available at 10.1186/s13065-023-01071-5.

## Introduction

Water contamination has become a global challenge to human survival. The desire for needs including water, food, agricultural goods, and other natural resources has grown as a result of industrial development, urbanisation, and an increase in world population [1, 2]. Pollution of water resources has grown in developing nations as a result of increasing agricultural and industrial activity as well as inappropriate industrial wastewater discharge into the environment [3, 4]. Non-biodegradable heavy metal cations (Pb^2+^, Cd^2+^, Hg^2+^^,^ Zn^2+^, etc.) and As anions (arsenate AsO_4_^−3^, arsenite AsO_3_^−3^) accumulate in the human body and can cause serious health problems [5, 6]. Human activities, including mining, electroplating, metallurgy, and heavy chemical industries, contribute immensely to the release of heavy metals, e.g. lead, cadmium, copper, etc., into the ecosystem, posing severe risks to ecosystems and human health [7]. Heavy metals are hazardous because of their non-biodegradability, being highly toxic, and associated with bioaccumulation (they can easily concentrate in living organisms), thus causing long-term danger to humans and other species through the food chain, thus increasing the chances of various human diseases and disorders like cancer, liver, and kidney damage, and hypertension [8, 9]. For instance, Lead (Pb) exposure, particularly at high concentrations, has the potential to result in anaemia, sluggishness, brain damage, and even death [10]. Cadmium (Cd) is a toxin and cancer-causing contaminant [11]. Exposure to Nickel (Ni) toxicity, has been found to have skeletal abnormalities, chromosomal abnormalities, carcinogenic development, impaired endocrine functions, the emergence of neurological disorders (such as homonymous hemianopsia), GIT disorders with the symptoms of nausea, vomiting, and diarrhoea among others, and the onset of metabolic disorders with a variety of symptoms and indications of metabolic stress [12, 13].

The World Health Organization has specified the maximum permissible quantities of Cd^2+^ and Pb^2+^ in drinking water at 0.003 mg/L and 0.01 mg/L, respectively [14, 15]. To improve the state of the ecological environment and to protect human health, it is crucial to remove dangerous heavy metal ions from contaminated water. Steroid hormones have emerged as a class of potential environmental pollutants of concern in recent decades, owing to their detection in the aquatic environment and their potential adverse physiological consequences in species and water bodies [9]. In aquatic animals, and humans, both naturally derived and synthetic steroid hormones, have potential to interrupt normal hormonal functions such as development, metabolism, and other bodily functions or processes, especially during crucial developmental periods [14]. Steroid hormones are not totally eliminated during conventional wastewater treatment, according to literature reports, and are eventually discharged into the aquatic environment [15]. Exposure to wastewater effluent containing steroid hormone concentrations as low as 0.1–4.2 ng/L can disrupt the normal functionality of the endocrine systems of fish and wildlife, thereby disrupting reproduction and development [16], and male feminisation and vitellogenin production occur as a result, reducing the rate of egg fertilisation [17]. Only a few researchers have delved into the human health risks of being exposed to natural and synthetic steroid hormones in the environment. There could be a connection between the 17-α estradiol (E2) metabolite 16-hydroxy-E2 and breast cancer, as well as an elevated risk of liver adenomas and hepatocellular carcinomas linked with long-term usage of 17-α ethinylestradiol (EE2) [18]. Increased amounts of androgens may contribute to the pathogenesis of prostate cancer, according to epidemiological observations [19]. Moreover, investigations have revealed that prepubescent children who are exposed to low levels of testosterone develop early puberty [20]. Other studies concluded that environmental sources of steroid hormones posed little harm to people when compared to typical body oestrogen concentrations, based on worst-case exposure estimations [21]. Several researchers have reported the occurrence of steroid hormones among the emerging contaminants found in wastewater treatment plants (WWTPs) [22–25].

Many researchers have reported the use of biochar in the treatment of emerging contaminants (ECs) in water [26–28]. However, due to the limitations associated with the use of raw biochar, such as low adsorption capacity, limited adsorption range, and other defects, the biochar must be modified to enhance its removal efficiency.

To safely remove contaminants from water, some methods including electrochemical treatment [29], chemical precipitation [30], solvent extraction [31], ion exchange [32], Photocatalysis [33], membrane filtration [34], reverse osmosis [35], and adsorption [36] have been used. Adsorption is regarded as an interesting and efficient approach among these technologies because of its flexibility, simplicity of operation, and relatively inexpensive. In the decontamination of pollutants, a variety of common functional materials, such as natural mineral materials and synthetic nanomaterials, have been extensively employed. However, the key difficulty remains the design of a cost-effective and long-lasting adsorbent.

Biochar is a carbon-rich product resulting from the thermal treatment of biomass and other wastes (such as crop and wheat straw, wood biomass, waste sludge, chicken manure, and other materials) in a low-oxygen chamber [25]. Owing to its vast potential in practice and fascinating properties, such as its large surface area, porosity, and the presence of various surface functional groups with good abilities to adsorb toxic contaminants, biochar is regarded as a promising adsorbent for the removal of water contaminants when compared to typical commercially produced activated carbon [37, 38].

However, there are several inherent disadvantages of using pristine biochar, such as the limited functional groups on the surface and their large particle size, which restrict their removal efficiency in environmental remediation and make it more difficult to use them to remove contaminants in heavily contaminated water. To overcome the limitations of using raw biochar, some chemical modification approaches have been explored to improve the performance of biochar, notably acid and alkali activation, metal salt activation, and loading nanoparticle materials onto the biochar [17–20]. For example, It was found that biochar produced by pyrolysis and activated with KMnO_4_ had a considerably enhanced capacity to remove heavy metals (HMs) from aqueous solutions when compared to its pristine biochar counterparts [25, 39].

In another investigation, an exclusive hydrothermal chemical approach was used to produce manganese ferrite/biochar composite materials, and these composites performed well when applied for the removal of heavy metals in wastewater [40]. Although most chemical modification methods for obtaining effective biochar-based composite materials are relatively simple, they still have some disadvantages, including high cost, the need for harsh experimental conditions (i.e., strict control of pH, pressure, and temperature), and the use of toxic chemicals in large quantities. Furthermore, for biochar to be utilised in engineering applications, it must have a larger surface area and a smaller particle size [41]. Thus, a green, sustainable, and low-cost approach to generating functional biochar for the practical removal of heavy metals and steroid hormones is highly sought-after.

Ball milling, a mechanochemical process that uses mechanical energy to induce structural and chemical changes in materials, is an emerging solvent-free approach for the development of modified biochar and its derivatives due to its cost-effectiveness, ease of usage, and ability to scale up [42]. The idea is that the biochar may be crushed into nano-sized particles by passing it through the grinding balls in the grinding jar inside the planetary ball mill [43]. It has been reported that ball milling can also enhance the interior pore network of biochar, thereby significantly boosting the specific surface area and adsorption capacity [44].

To the author’s knowledge, the applicability of ball-milled biochar derived from African Star Apple seed shell for the removal multi steroid hormones and heavy metals has not been published; to this end, the objectives of this research are: (1) To produce, modify and characterise the biochar derived by carbonization of African Star Apple seeds shell; (2) to examine the sorption of heavy metals and steroid hormones by ball-milled biochar in batch adsorption tests under numerous environmental conditions and evaluate the results using kinetic and isotherm models; (3) to determine the efficiencies of the modified biochar in the removal of heavy metals and steroid hormones in wastewater.

## Materials and methods

### Materials

All chemical reagents used for this work were all analytical grade purchased from Sigma-Aldrich with a purity of (≥ 98%): the cadmium nitrate Cd(NO_3_)_2_·4H_2_O), lead nitrate Pb(NO_3_)_2_, and nickel nitrate Ni(NO_3_)_2_·6H_2_O. Metal stock solutions of 1 000 mg L^−1^ were made by dissolving precisely weighed masses of each constituent in a 250 mL volumetric flask and filling up to mark using ultrapure water. To avoid metal ion hydrolysis, the standard stock solution was moderately acidified. For additional tests, working standard solutions were made by diluting the standard stock solutions with ultrapure water. The pH of the standard solution was controlled by adding solutions of 0.1 M NaOH or HNO_3_. Accurate weighing was done using an analytical balance (Mettler Toledo, Model AG204, Switzerland) with a precision of 0.0001 g. Estriol (C_18_H_24_O_3,_ 97% purity, molecular weight of 288.38 g/mol), β-Estradiol (C_18_H_24_O_2_, 98% purity, molecular weight 272.38 g/mol), α-Estradiol (C_18_H_24_O_2_, 98% purity molecular weight 272.38 g/mol), Testosterone (C_19_H_28_O_2_, 99% purity molecular weight 288.42 g/mol), Progesterone (C_21_H_30_O_2_, 99% purity molecular weight 314.46 g/mol) and Bisphenol A (C_15_H_16_O_2_, 99% purity molecular weight 228.29 g/mol). All stock solutions of steroid hormones were prepared by dissolving 10 mg powder of each standard into a 10 mL standard flask containing 1 mL 50/50 methanol and ultrapure (UHP) water (18.2 Ω/cm) and made up to the mark.

### Adsorbent preparation

African Star Apple seeds shell, the feedstock used for this study, were washed with ultrapure water, dried inside the oven at 80 °C for 4 h, and stored in an airtight pack before the carbonization. The biochar was produced according to the method reported [45]. A 20 g sample of the dried material was placed in the muffle furnace at temperatures 350 °C 450 °C, and 550 °C at a heating rate of 10 °C/min for 1 h in a limited supply of oxygen. The black solid residue obtained was ground by mortar and pestle to produce dried biochar of small sizes. Each produced biochar was then kept airtight and labelled by their carbonization temperatures, namely ASA350, ASA450, and ASA550 before the characterisation and further modification.

The biochar produced by carbonization was further modified via the mechanochemical method using a Retsch MM 200 mixer mill machine. A 1.2 g sample of each biochar (ASA350, ASA550, and ASA550) was weighed into each ball milling jar (80 mL) containing 120 g beads (balls) in the ratio of 1:100 biochar/ball mass ratio [46]. The rotational speed was set at 850 rpm for a total milling time of 6 h with the rotational direction changed every 2 h, and the ball-milled biochar was thereafter obtained and was labelled as BASA350, BASA550, and BASA550, respectively.

### Characterisation of adsorbents

In this study, the Barrett–Joyner–Halenda (BJH) method was used to determine pore volume and pore size, and surface area, was determined using Brunauer–Emmett–Teller (BET) analysis (Micromeritics® ASAP™ 2020 with TriStar™ II version 2.00 software, Micromeritics Instrument Corporation, Norcross, GA, USA). The crystallinity of the biochar was analysed using X-ray powder diffraction (Rigaku SmartLab X-Ray diffractometer (XRD) with Cu Kα radiation (wavelength λ = 0.154 nm) (Rigaku Corporation, Tokyo, Japan). The surface functional groups present on the biochar were identified using Fourier transform infrared (FTIR) spectroscopy (4000–400 cm^−1^ wave number) (Vertex 70v FT-IR Spectrometer, Bruker Optics, Billerica, USA). An energy-dispersive spectrometer and a scanning electron microscope (SEM) were used to analyse the surface morphology, structure, and elemental content of the biochar. The SEM used was a JEOL JSM-IT 300 SEM (JEOL Ltd., Tokyo, Japan) coupled with an Oxford Instruments EDS system (Oxford Instruments, Abingdon, Oxfordshire, UK). Thermogravimetric analysis of the biochar was carried out at a heating rate of 20 °C/min, using the TGA/DSC SDT Q600 thermogravimetric analyser with TA software (TA Instruments, New Castle, Delaware, USA). An X-ray photoelectron spectrometer (XPS) (Thermo Fisher Scientific™ X-ray Photoelectron Spectrometer (XPS), Waltham, MA, USA) was used as a surface analysis technique to determine the elemental composition and oxidation states of elements at the surface of the biochar. The Raman spectra of the biochar were obtained using a confocal Raman microscope (XploRA™ Plus, Horiba Scientific, Kyoto, Japan) with laser energy of 2.411 Ev.

### Batch adsorption experiment

The removal of the heavy metals by pristine and ball-milled biochar was assessed by trial experiment. Briefly, the removal percentage of pristine biochar (ASA350, ASA450, and ASA550) and that of the modified biochar produced by ball milling (BASA350, BASA450, and BASA550) were evaluated by adding 20 mg of each adsorbent to the 25 mL solution containing 2 mg/L mixture of all the heavy metals under investigation (temperature = 25 °C, pH = 6.5, contact time = 60 min). The solutions were then filtered by a PVDF syringe filter of 0.45 μm pore size and 25 mm diameter, and the filtrates were collected in ICP-OES plastic sample tube vials and were analysed. The adsorbent (BASA550) with the highest removal efficiency which was also confirmed by the results obtained from the characterisation of the biochar was selected for further optimisation of the adsorption experiment. The wastewater application was carried out by spiking the wastewater sample with the prepared synthetic solution of 2 mg/L solutions of the mixed solutions of the heavy metals and steroid hormones with the addition of 20 mg of BASA550.

After each experiment, the solutions were then filtered by a PVDF syringe filter of 0.45 μm pore size and 25 mm diameter. The filtrates were then collected into ICP-OES and HPLC vials and analysed accordingly. The results obtained were then used for adsorption isotherms and adsorption kinetics to determine the equilibrium of the adsorption reaction.

Equation (1) was employed for the determination of the percentage removal of heavy metals and steroid hormones and Eq. (2) [47] to determine the amount of pollutants adsorbed by the adsorbent at equilibrium (*q*_e_) in mg/g [48].

1 $$\text{Removal}\; \text{efficiency}\; {\%}=\left(\frac{\text{C}_{\text{o}}-{\text{C}}_{\text{e}}}{{\text{C}}_{\text{o}}}\right)\times 100$$

where *C*_o_ and *C*_e_ are the initial concentrations of pollutants and the concentrations of pollutants at equilibrium, respectively (mg/L).

2 $${\text{q}}_{\text{e}}= \left(\frac{\text{C}_{\text{o}}-{\text{C}}_{\text{e}}}{\text{m}}\right)\times \text{V}$$

where *C*_o_ and *C*_e_ are the initial concentrations of pollutants and the concentrations of pollutants at equilibrium, respectively (mg/g), *V* is the volume of the solution (L), and *m* is the mass of the adsorbent (g).

## Results and discussion

### Characterization of the material

#### BET of the pristine and modified biochar

**Fig. 1:**
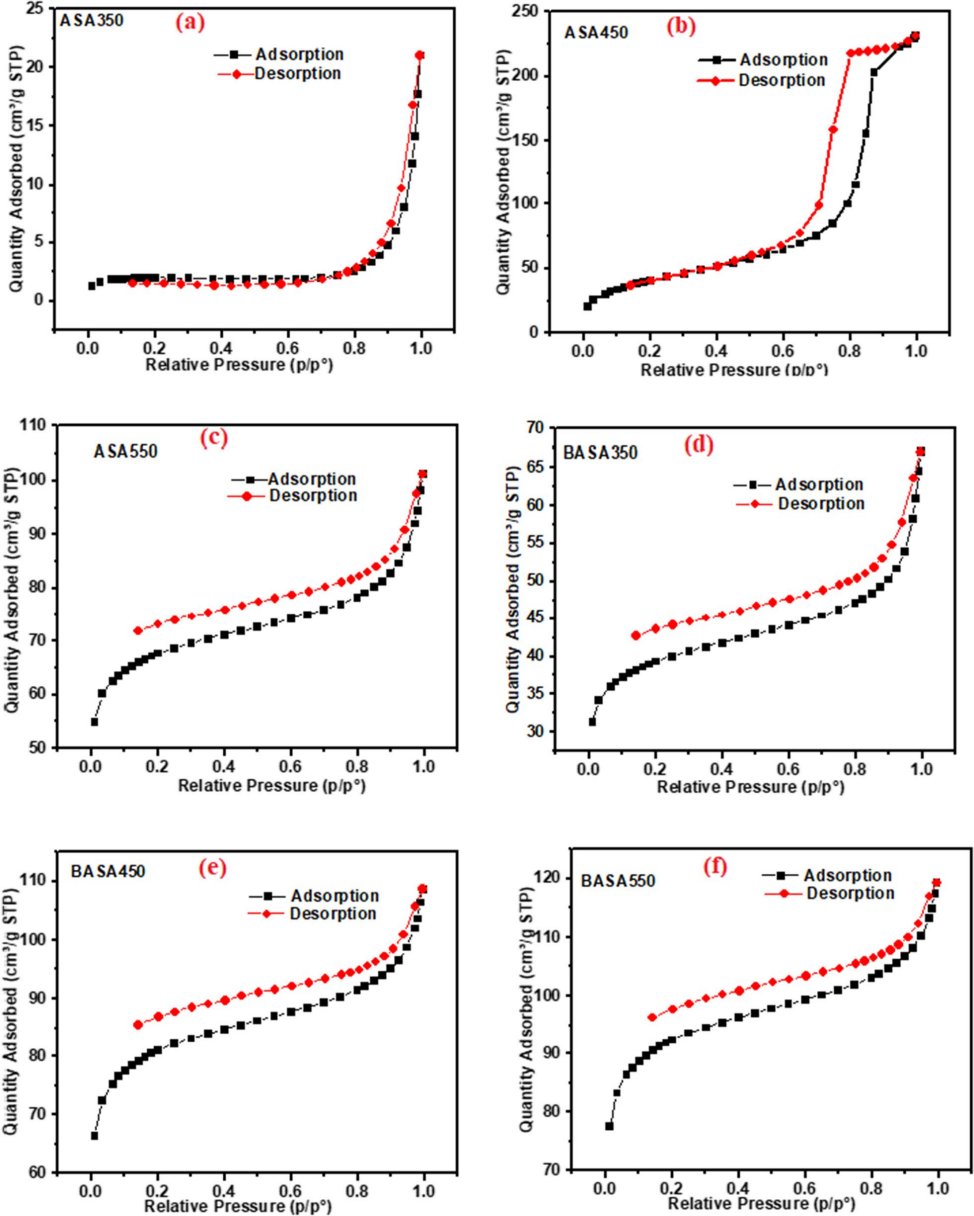
The isotherm curves for adsorption and desorption of **a** ASA350, **b** ASA450, **c** ASA550, **d** BASA350, **e** BASA450 and **f** BASA550

The surface properties of the adsorbents (pristine and ball-milled biochar) used for this study were characterised by BET analysis and are shown in Table 1. The surface areas of biochar that were produced were temperature-dependent. The surface areas of biochar were found to increase with the increase in carbonization temperature. The pristine biochar produced by carbonization at 350 °C (ASA350) was found to have a surface area of 88.79 m^2^/g which increased to 122.63 m^2^/g for the pristine biochar produced by carbonization at 450 °C (ASA450), while the biochar obtained at a temperature of 550 °C (ASA550) was found to have the highest surface area of 172.92 m^2^/g. The significant increase could be attributed to the increase in the carbonization temperature of the biochar which resulted in the opening of pores of the carbon and other mineral contents in the material [49]. The grain size of the pristine biochar is much larger than that of the ball-milled biochar. The ball milling process significantly reduced the grain size of pristine biochar from 67.57 nm to 19.84 nm of the ball-milled biochar; this was due to the high energy impact of grinding balls with biochar in the grinding jar during the ball milling process. Ball milling also enhanced the surface area and pore volume of the ball-milled biochar, the surface area (SA) of BASA350 (104.28 m^2^/g) was found to increase by 20% in comparison to that of the pristine biochar ASA350 with SA of 88.79 m^2^/g. The pore volume of ball-milled biochar BASA550 (0.080 cm^3^/g) was also found to be six times higher than that of the pristine biochar ASA550 (0.013 cm^3^/g) produced by carbonization at 550 °C. This finding demonstrated that ball-milling not only enhanced the exterior surface area of the grains by reducing the particle size but also increased the interior surface area by opening up the inner pore structure [50]. Furthermore, in Fig. 1, the type I adsorption isotherm curve was seen in the BASA550, that the ball-milled biochar is predominantly mesoporous within the pore size range of 2–50 nm with an average pore diameter of 6.22 nm and the total pore volume of 0.080 (cm^3^/g) for BASA550 [51].

**Table 1:** Surface properties of biochar and ball-milled biochar

Adsorbent (pristine and ball-milled biochar)	Surface area S_BET_ (m^2^/g)	BJH pore volume (cm^3^/g)	BJH pore size (nm)	Grain size of pristine and ball-milled biochar (nm)
ASA350	88.79	0.016	3.06	67.57
ASA450	122.63	0.010	3.75	48.92
ASA550	172.92	0.013	3.57	34.70
BASA350	104.28	0.053	5.15	57.53
BASA450	249.62	0.079	5.09	24.04
BASA550	302.35	0.080	6.22	19.84

*BJH* Barrett–Joyner–Halenda approach

#### XRD analysis

**Fig. 2:**
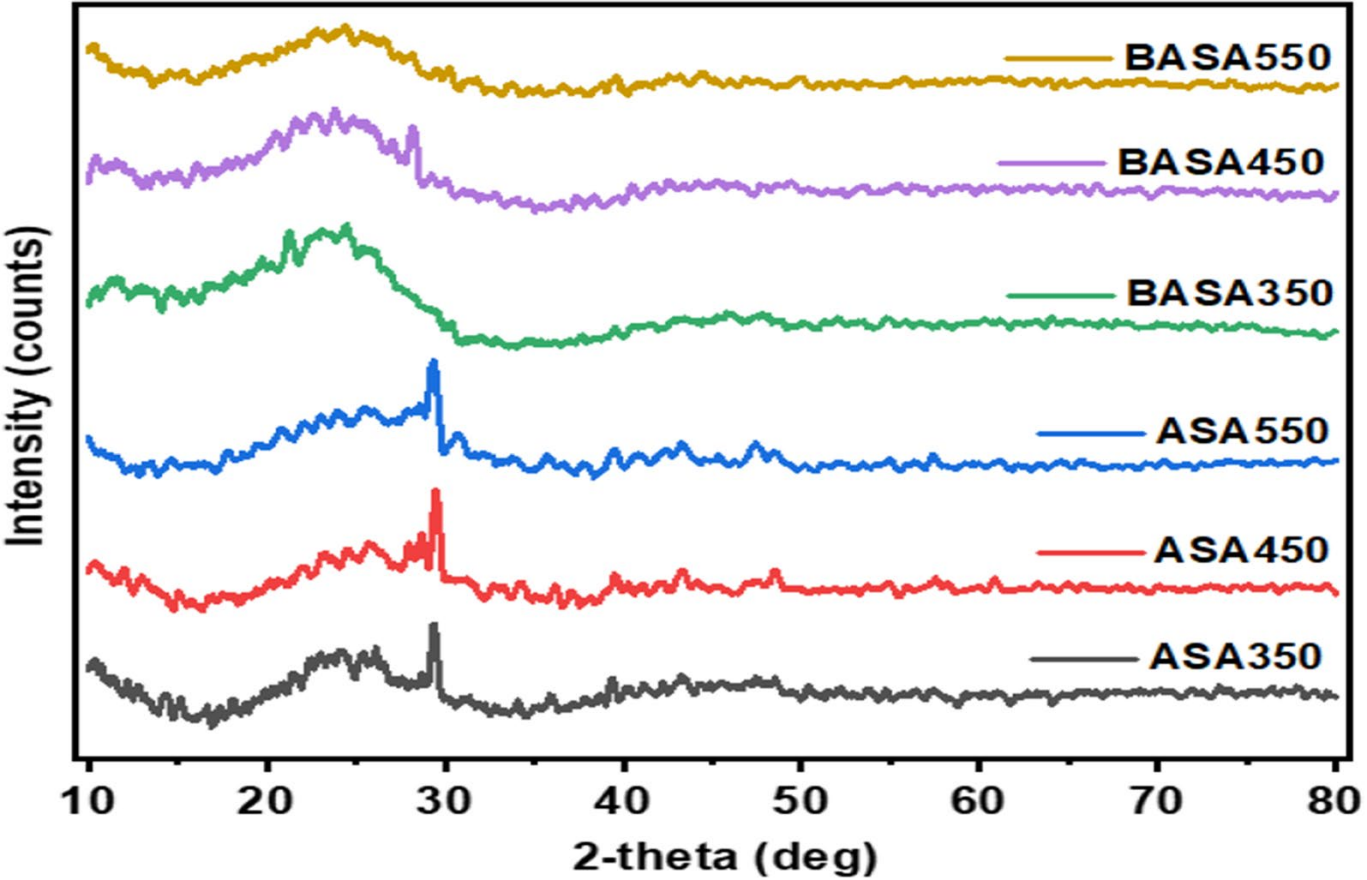
XRD spectra of the pristine and ball-milled biochar obtained at 350, 450, and 550 °C

Figure 2 depicts the XRD spectra of the raw biochar obtained from African star apple (ASA) waste material at different temperatures, 350 °C, 450 °C, and 550 °C (ASA350, ASA450, and ASA550, respectively), and those of the ball-milled modified biochar (BASA350, BASA450, and BASA550). The absence of sharp long peaks in the spectra indicated that the ball-milled biochar was completely amorphous, while the raw biochar was also amorphous but there is the presence of short peaks around 28° which disappeared in the spectra of ball-milled biochar. This could be because the ball milling operation reduces the biochar sizes in the cylindrical grinding jar in the presence of grinding balls as confirmed by BET analysis (Table 1). The broad peaks between 23° and 27° appeared in all the raw and the ball-milled modified biochar and could be attributed to the presence of carbon which is the dominant element in the biochar [52]. The small peaks between 2θ 40°–50° correspond to nanographene structures which are commonly present in carbon materials as well as in biochar material [53].

#### FTIR analysis

**Fig. 3:**
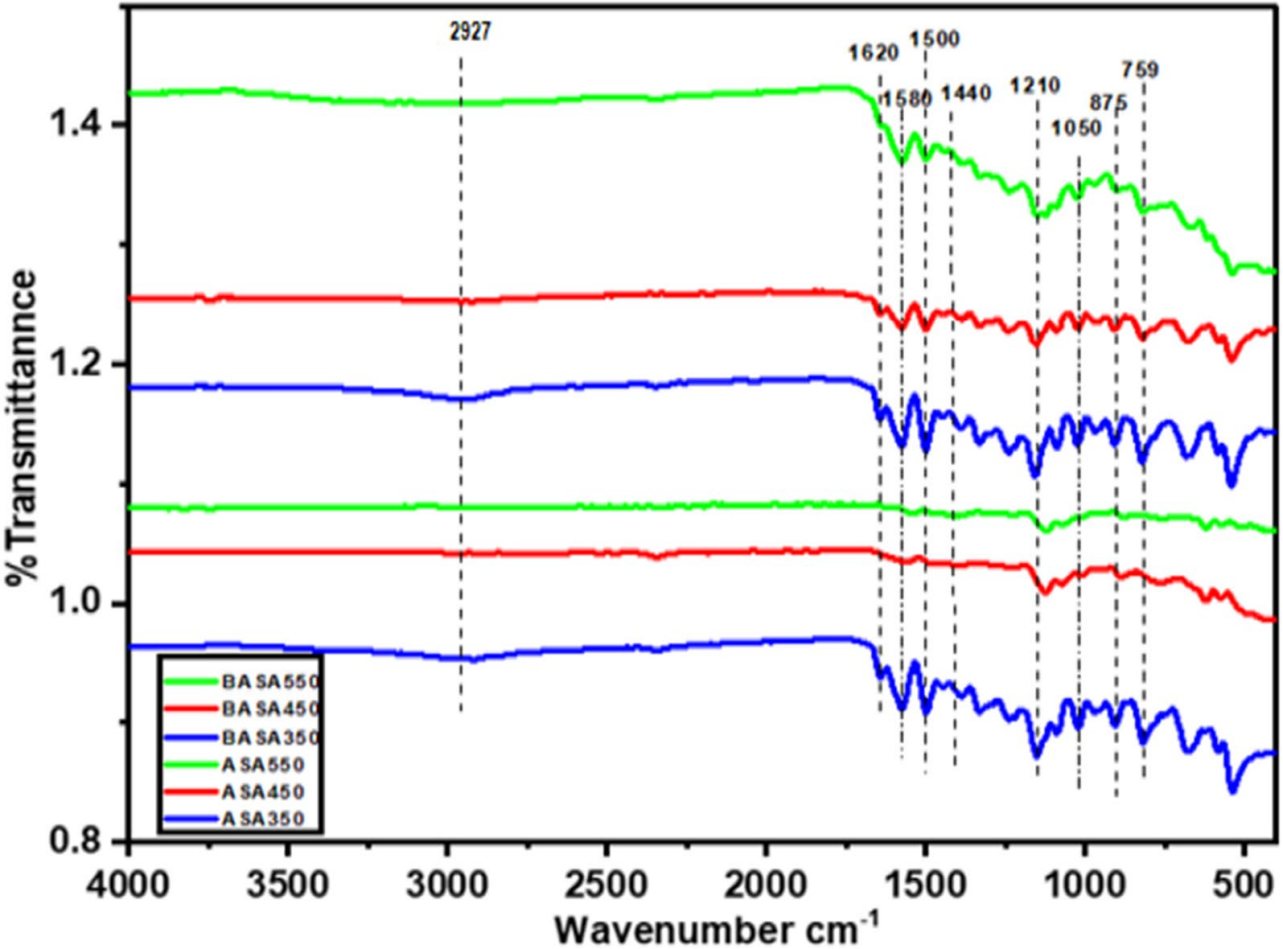
FTIR spectra of raw and ball-milled biochars

Fourier transport infrared (FTIR) was used to identify the various surface functional groups present in the raw and the ball-milled biochar. Similar peaks can be observed in all the biochar spectra in Fig. 3 except the C–H peak of aliphatic stretching vibration at 2 927 cm^−1^ which is more pronounced in the ASA 350 spectra obtained at a temperature of 350 °C, which relatively disappeared in the biochar obtained at elevated temperatures (450 °C and 550 °C) could be attributed to the thermal decomposition of the alkyl-CH_3_ groups which are more present at a lower temperature [54]. The peak at 1 620 cm^−1^ could be attributed to C=O stretching and C=O vibration of carbonyl groups of lignin [52]. The peak at 1 580 cm^−1^ represents C=O aromatic stretching vibration. The peak at 1 050 cm^−1^ is linked to the sp^3^ hybridisation of carbon atoms present in biochars [52], and the peak at 875 cm^−1^ reveals the aromatic C–H bonds in the fingerprint region, and the C–H bending of alkynes can be seen in the peak at 759 cm^−1^ [52].

#### SEM and EDS analysis

**Fig. 4:**
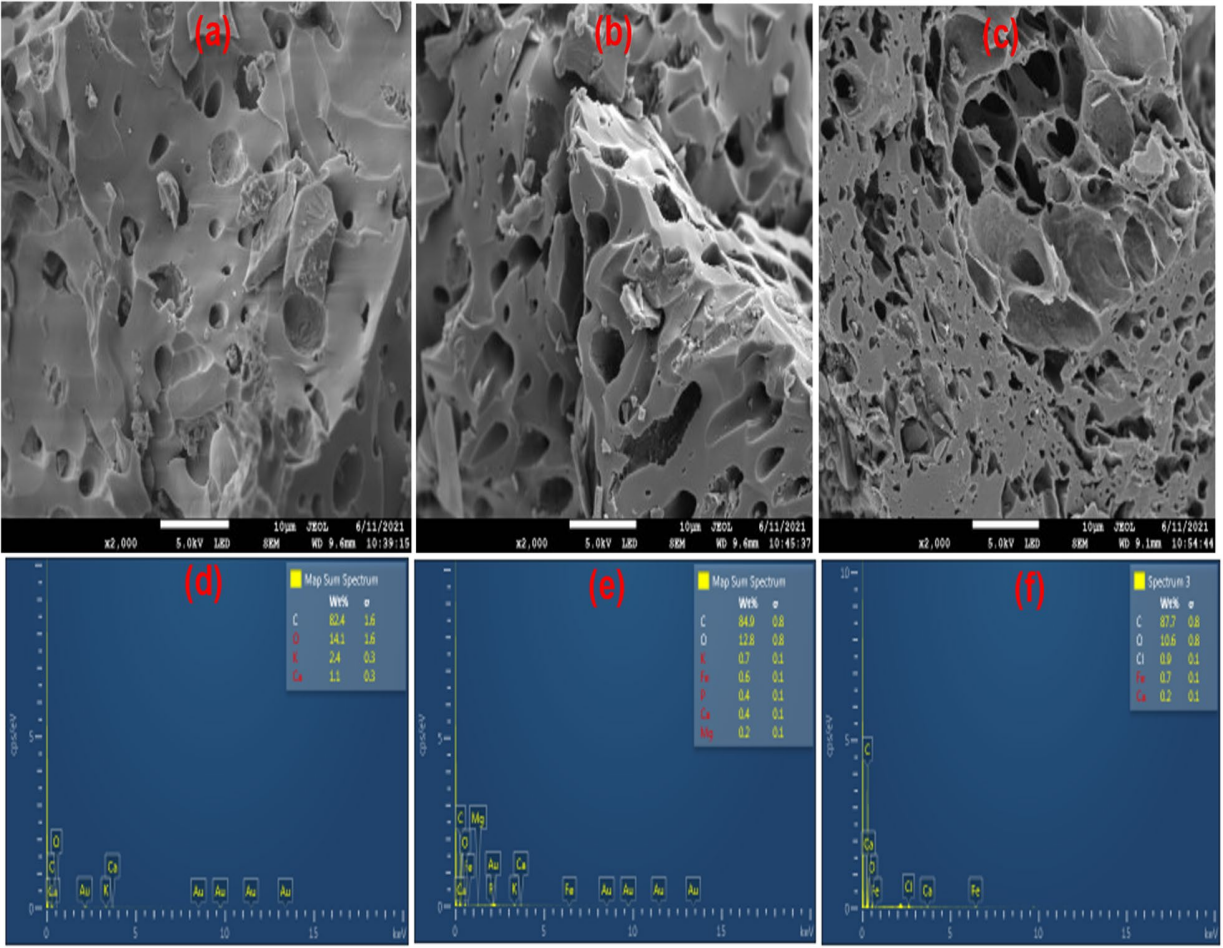
SEM micrographs and EDS mapping images of ASA550

**Fig. 5:**
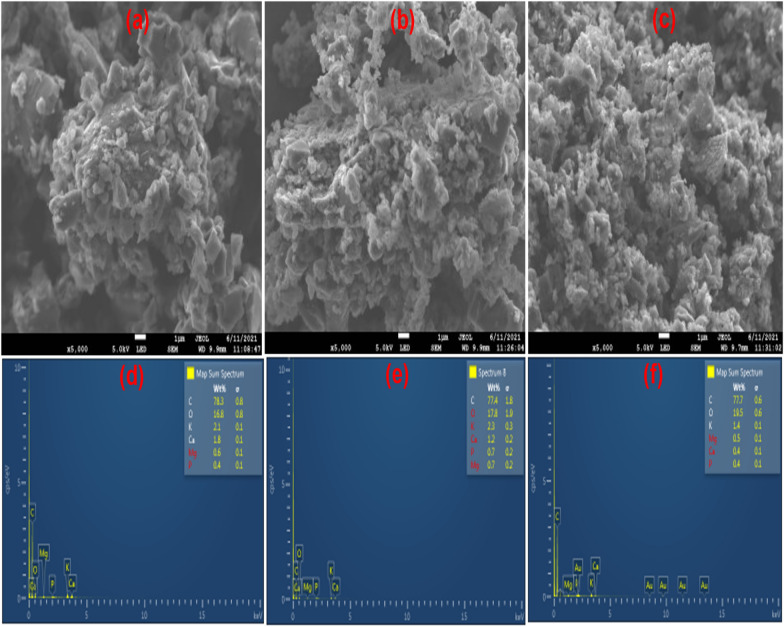
SEM micrographs and EDS mapping images of BASA550

The surface morphology, textural properties, and elemental composition of raw (ASA) and ball-milled biochars (BASA) were analysed by high-resolution scanning electron microscope (SEM), JEOL JSM-IT 300 (JEOL Ltd., Tokyo, Japan) equipped with Oxford Instruments EDS system (Oxford Instruments, Abingdon, Oxfordshire, UK). The SEM micrographs and EDS mapping images in Fig. 3 reveal the porous surface of the raw biochar with pores. The pores increased as the carbonization temperature increased from 350 to 550 °C for ASA450 and the pores became larger for ASA550; this could be attributed to the opening of the pores when the temperature increases which thermally degrade other volatile organic compounds present in the biochar thereby opening pores on the surface of the biochar. The SEM images of ball-milled biochar can be seen in Fig. 5; the ball-milling operation made the biochar more porous and formed cluster nanoparticles after ball milling. The ball milling reduced the particle size of the ball-milled biochar; the raw biochars are on a larger scale (10 µm) in Fig. 4a–c for ASA550, while the ball-milled biochar is on a smaller scale (1 µm) in Fig. 5a–c. The decrease in the particle size of the ball-milled biochar (BASA550) confirmed that ball milling affects both the internal and external surface of the biochar, which in turn increases the surface area of the ball-milled biochar, which is good as an adsorbent [45, 55]. The EDS mapping images of the raw biochar (ASA550) in Fig. 4d–f and of the ball-milled biochar in Fig. 5d–f revealed the elements and their composition in the biochar at different temperatures and after ball milling (BASA550). Both raw and ball-milled biochar have similar elemental composition; the slight difference could only be seen in the percentage of oxygen in the composition. The oxygen content of the raw biochars increased after ball milling; this could be attributed to an increase in the number of oxygen-containing surface functional groups during the ball milling process [56].

#### TGA studies

**Fig. 6:**
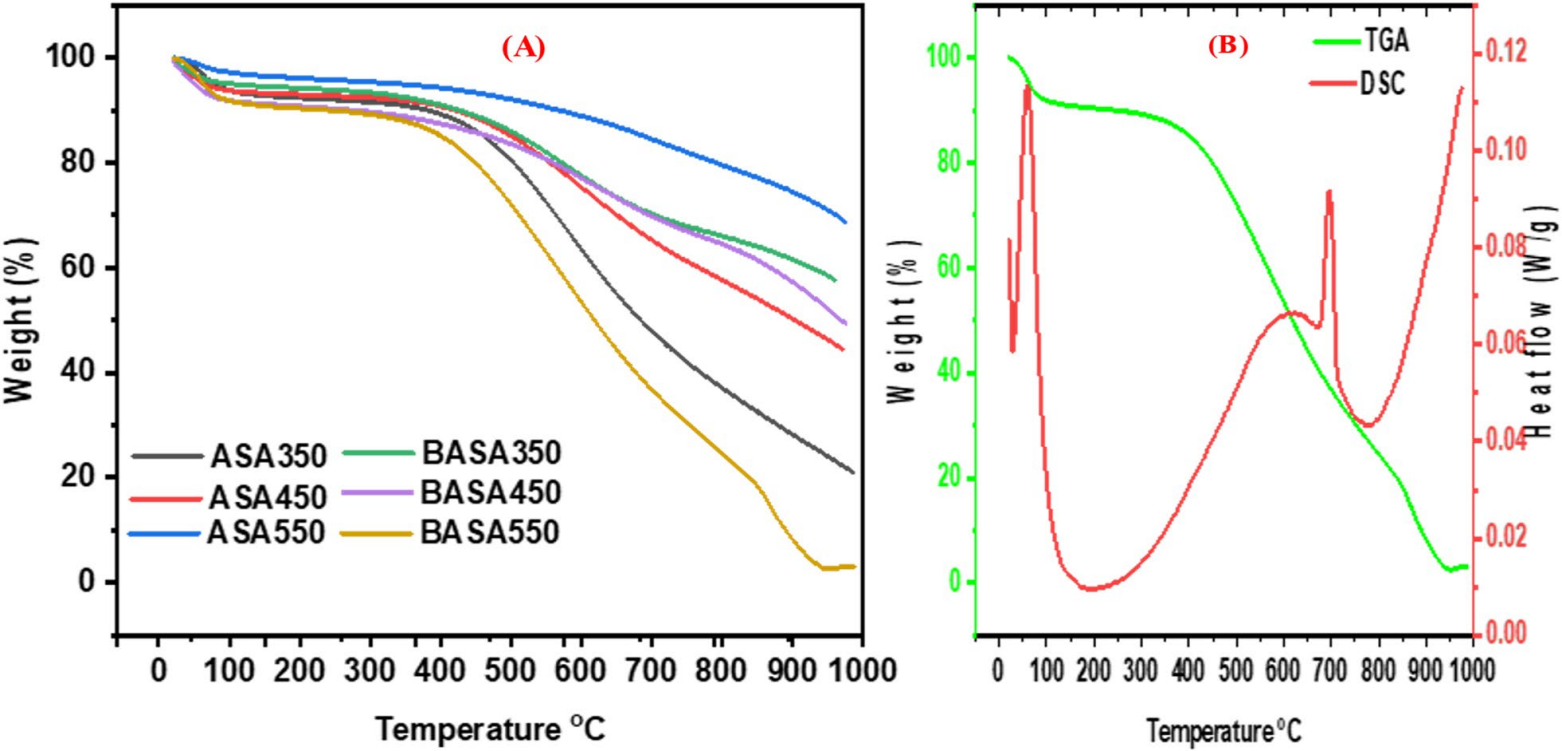
Thermal analysis (TGA and DSC) of the ball-milled biochar

Figure 6A shows the thermal properties of the biochar (raw and ball-milled) when subjected to thermal treatment. The slight drop on the chart at a temperature between 20 and 100 °C represents the water loss and loss of other volatile organic components present in the biochars. The second significant disintegration occurred around 470 °C with only a 20% loss of weight. The biochar sample was thermally stable up to a temperature of 700 °C before further breakdown. Figure 6B depicts the TGA and DSC curves of ball-milled biochar (BASA550). The green line on the chart represents the TGA curve while the red line on the chart is for the DSC curve. As shown on the graph, two major peaks correspond to exothermic processes. The first exothermic peak can be seen at a temperature of 100 °C, which is the heat loss that corresponds to the weight loss due to the water and some other organic substances in the sample. The second exothermic peak at a temperature of around 700 °C resulted in about 50% loss of the weight of the sample before the continuous disintegration and loss of weight above 700 °C. Both raw and ball-milled biochar are thermally stable within the temperature range of this experiment.

#### XPS analysis of ASA550 and BASA550

**Fig. 7:**
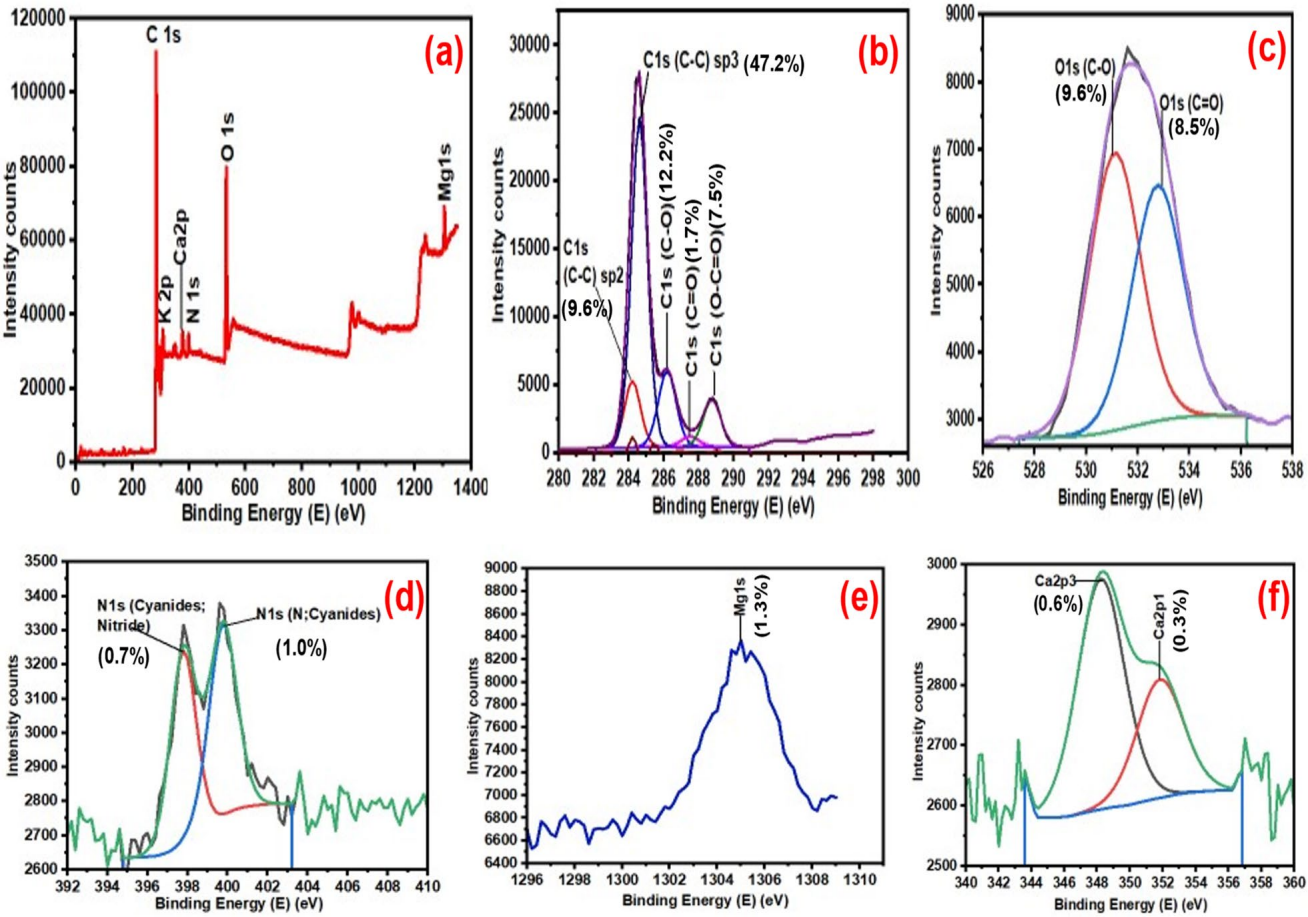
XPS spectra of ASA550: **a** full survey scan; **b** carbon; **c** oxygen; **d** nitrogen; **e** magnesium; and **f** calcium

**Fig. 8:**
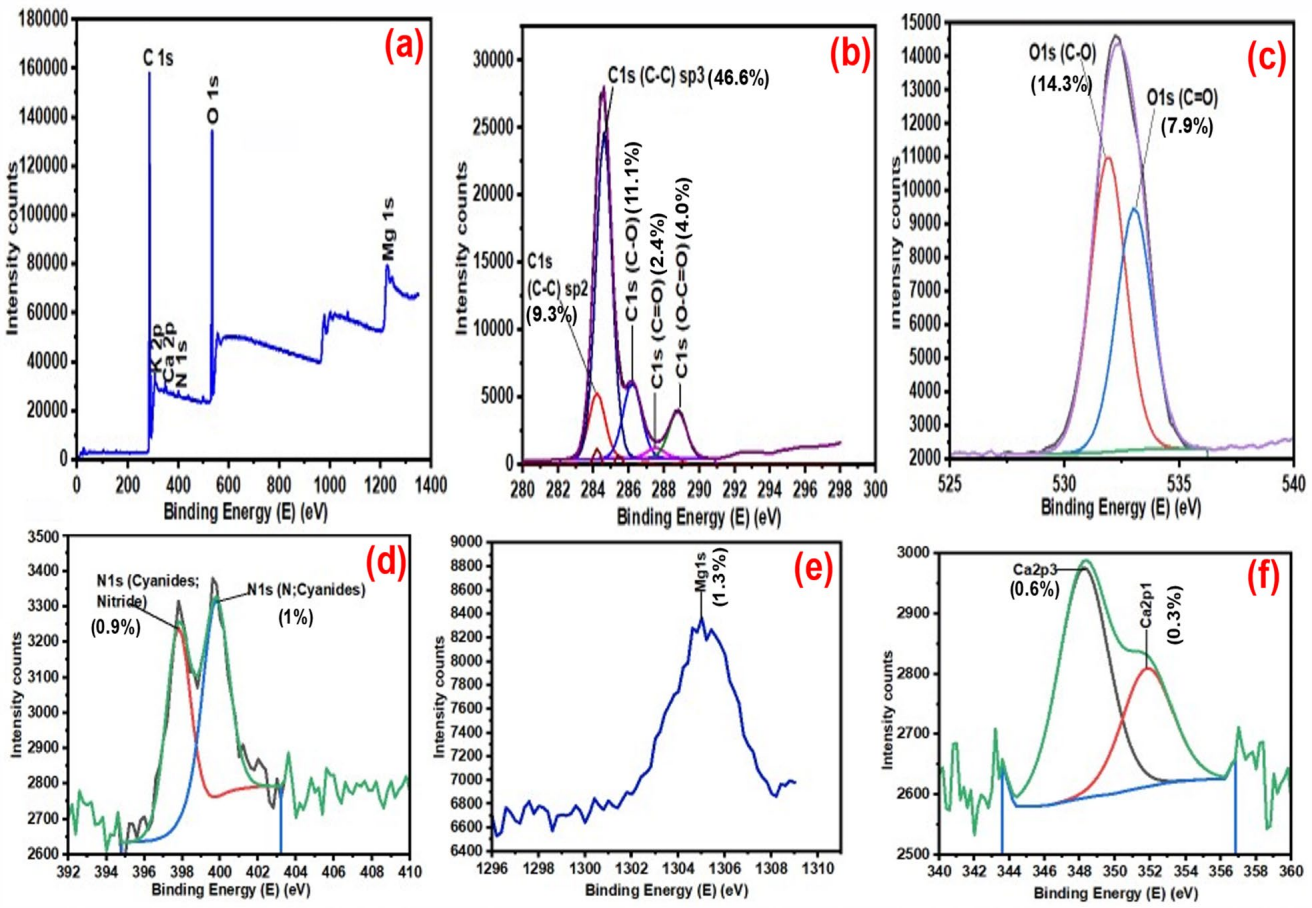
XPS spectra of BASA550: **a** full survey scan; **b** carbon; **c** oxygen; **d** nitrogen; **e** magnesium; **f** calcium

The elemental composition of the ASA550 and BASA550 with their respective percentage of occurrence and their oxidational states were studied using a Thermo Fisher Scientific™ ESCALAB 250Xi X-ray Photoelectron Spectrometer (Figs. 7 and 8). Figure 7a represents the full survey scan of ASA550 which revealed the presence of carbon, potassium, calcium, nitrogen, oxygen, and magnesium. As revealed in Fig. 7b, the carbon (C 1s) spectra of ASA550 revealed five peaks at C1 284.2 eV (9.6%) and C2 284.6 eV (47.2%) representing the C–C/C=C of graphitic or amorphous carbon, and C3 286.2 eV (12.2%), C4 287.5 eV (1.7%) are attributed to the sp^2^ hybridised carbon. The carbon C5 at 288.8 eV (7.5%) was related to the carboxyl functional group of (C=O) present in the raw biochar [57]. Figure 7c depicts oxygen (O 1s) spectra of pristine biochar with two distinct peaks of carbonyl oxygen in the carboxyl (COOH) group at the peak 531.4 eV (9.6%), while the second peak around 533.8 eV (8.5%) could be attributed to the hydroxyl (–OH) groups. Figure 7d shows nitrogen spectra with two peaks at 397.8 eV (0.7%) and at 399.8 eV (1.0%) which were attributed to nitride and cyanides, respectively. Figure 7e shows the magnesium spectra at a peak of 1305.1 eV (1.3%). Figure 7f shows the calcium spectra with peaks at 347.0 eV and 350.6 eV related to calcium carbonate (CaCO_3_). Figure 8a shows the full survey scan of XPS spectra for the BASA550. Similar functional groups were found in the spectrum of the ASA500 (Fig. 7a–f) and BASA550 (Fig. 8a–f). The significant differences in the spectra of the raw and ball-milled biochar can be seen in the total percentage composition of the carbon atom and oxygen atom. According to Fig. 7b, c of the ASA550, carbon accounted for 78.8%, and oxygen content was 18.1% in total. It can be seen in Fig. 8b, c that the total carbon content in the BASA550 decreased to 73.4% while the oxygen content increased to 22.2%, as shown in the XPS spectrum of the modified biochar. This change could be attributed to the effect of ball milling, and it confirmed that ball milling could induce oxygen functional (moieties) groups on the surface of the ball-milled biochar. The oxidation of oxygen with other carbon functional groups on the surface of the biochar during ball milling could account for this observation [56].

#### Raman spectra

**Fig. 9:**
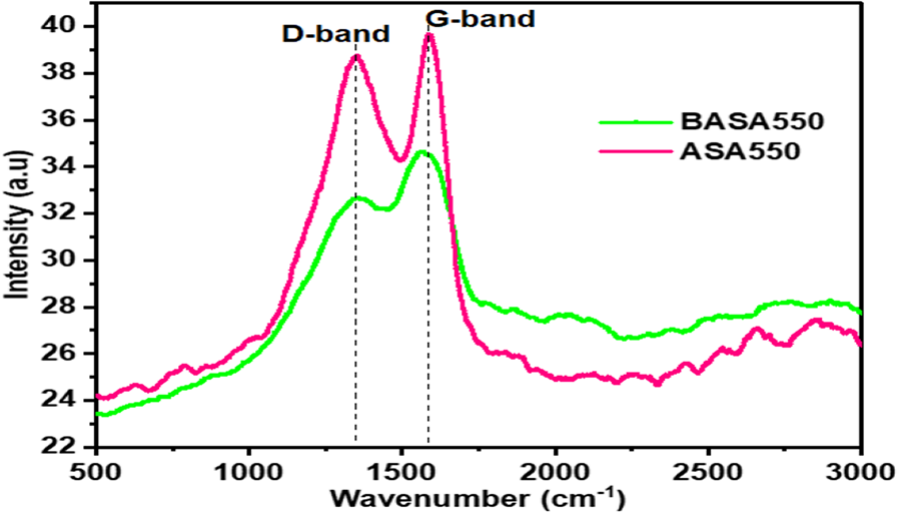
Raman spectra of ASA550 and BASA550

Figure 9 shows the Raman spectra of pristine biochar (ASA550) and ball-milled biochar (BASA550). The spectra of the ASA550 and BASA550 showed two separate well-defined bands which are popularly referred to as D and G bands. The in-plane resonance of the sp^2^ hybridised carbon atoms (G band) as well as the sp^3^ hybridised carbon atoms of disorderly graphite (D band) was ascribed to the Raman peaks at 1 350 cm^−1^ and 1 580 cm^−1^, respectively [58]. After ball-milling, the intensity of the D and G bands in pristine biochar decreased, which could be due to the disarrangement of the c-axis and the development of high specific surface area structures and is related to crystal size [59].

### Results of adsorption experiments

**Fig. 10:**
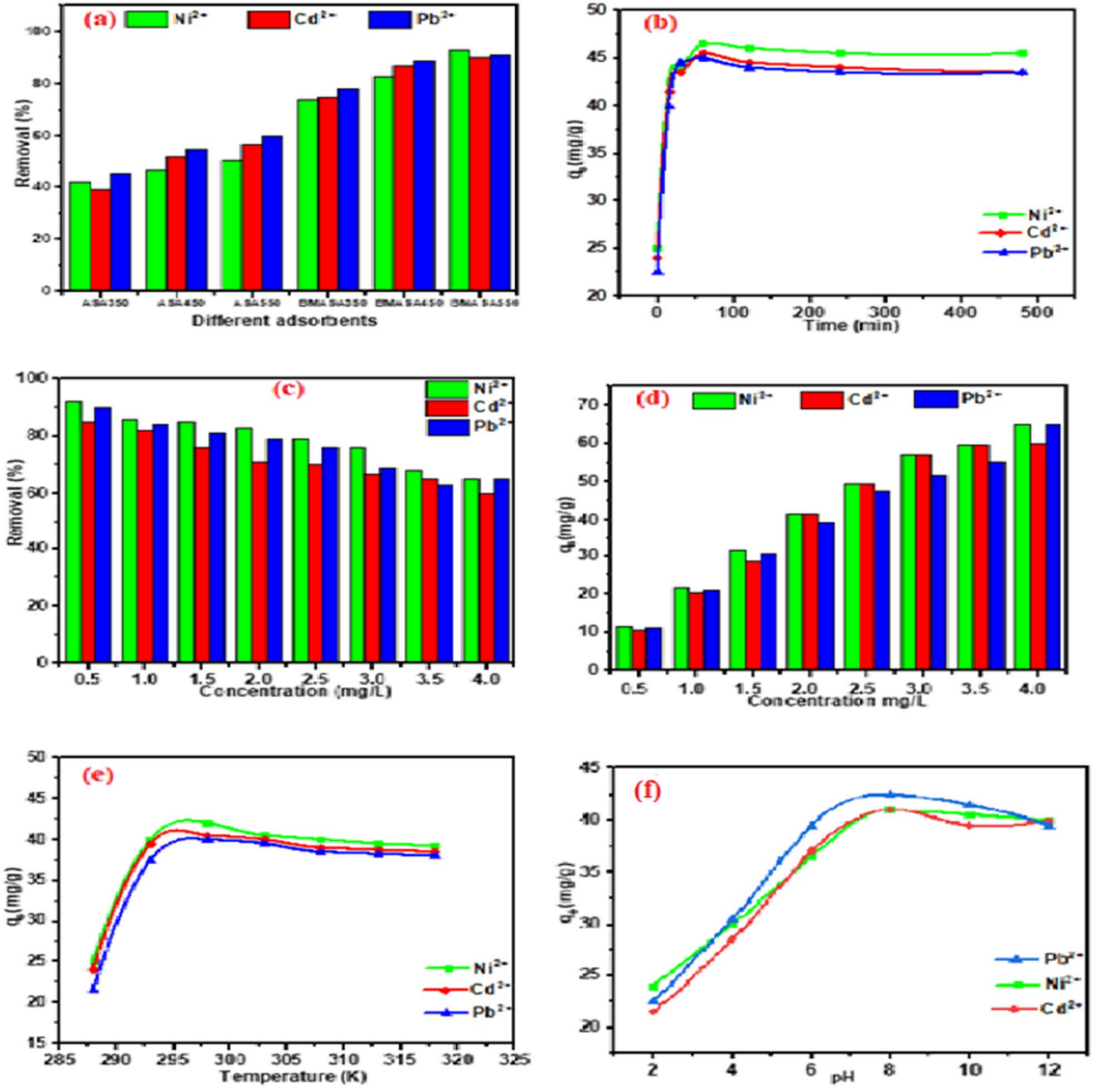
**a** Effect of adsorbent type; **b** effect of contact time; **c**, **d** effect of metal ion concentration; **e** effect of temperature, and **f** effect of pH on the adsorption of metal ions onto the surface of the BASA550

**Fig. 11:**
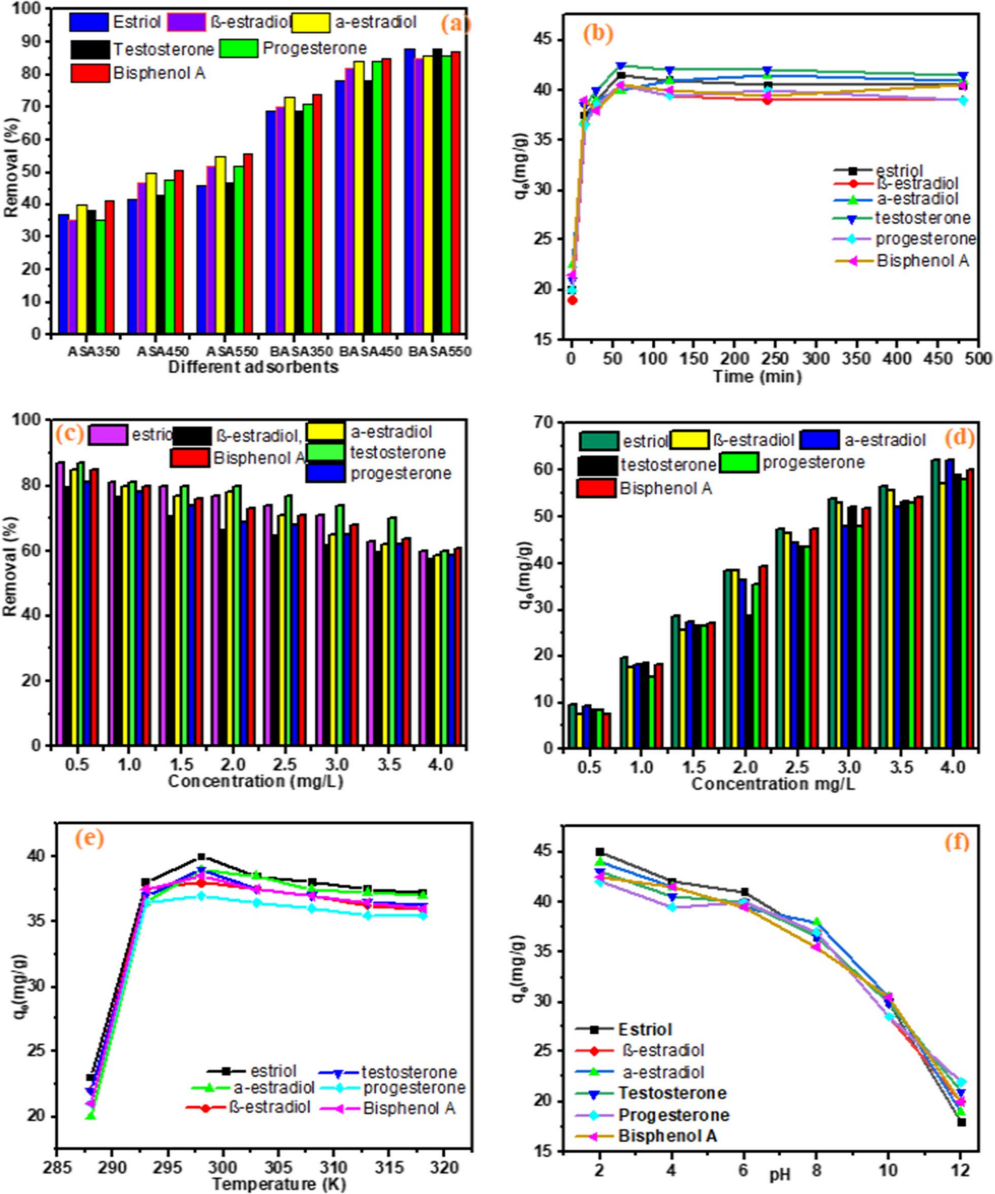
**a** Effect of adsorbent type; **b** effect of contact time; **c**, **d** effect of steroid hormones concentration; **e** effect of temperature, and **f** effect of pH on the adsorption of steroid hormones onto the surface of the BASA550

The results of the study on the metal ion removal percentage of pristine biochar (ASA350, ASA450, and ASA550) and those of the modified biochar produced by ball milling (BASA350, BASA450, and BASA550) are shown in Figs. 10a and 11a for the heavy metals and steroid hormones, respectively. From the result, the percentage removal increased slightly with the increase in carbonization temperature from 350 to 550 °C. This could be due to the opening of the pores of the biochar with an increase in the carbonization temperature; this was also confirmed by the BET result obtained in Table 1. The metal ion and steroid hormones removal percentage of ball-milled biochar obtained at 550 °C (BASA550) increased significantly after ball milling, with the highest percentage removal of 90%, 91%, and 93% for Cd, Pb, and Ni compared with the highest percentage removal of 56%, 63%, and 67% for Ni, Cd, and Pb for the pristine biochar obtained at the same temperature (ASA550). The highest percentage removal of 88%, 86%, 85%, 88%, 86%, and 87% compared with the highest percentage removal of 42%, 55%, 52%, 47%, 52%, and 56% for estriol, α-estradiol, β-estradiol, testosterone, progesterone, and Bisphenol A, respectively. The study of the effect of time in an adsorption experiment is very important to establish how fast an equilibrium could easily be attained. In this experiment, the study of contact time was done while keeping other parameters constant and varying the time (0–400 min) of the adsorption experiment. Figures 10b and 11b for the heavy metals and steroid hormones, respectively show that the adsorption capacity of the BASA550 increased steadily as the contact time between BASA550 and the three heavy metals and steroid hormones increased until the peak of the graph at 60 min indicating the optimum contact time. This could be explained by the increasing saturation of the active sites of the BASA550, which became saturated at a contact time of 60 min, after which the adsorption capacity was found to remain constant. This finding showed that the highest adsorption capacity of BASA550 for the removal of the Ni, Cd, and Pb (46.5 mg/g, 45.5 mg/g, and 45 mg/g, respectively) and estriol, α-estradiol, β-estradiol, testosterone, progesterone, and Bisphenol A (41.5 mg/g, 42.0 mg/g, 40.5 mg/g, 42.5 mg/g, 40.5 mg/g, 40.5 mg/g, respectively) was reached within 60 min of the adsorption process.

Figures 10c and 11c show the effect of the concentration of the solution of the heavy metal ions and steroid hormones on the adsorption capacity of BASA550. In this experiment, all the other variable parameters were kept constant, the heavy metal ion and steroid hormones concentration was varied from 0.5 to 4 mg/L with 0.2 g of BASA550. The results obtained showed that the percentage removal of the heavy metals and steroid hormones was favoured at lower metal ion and steroid hormone concentrations in the solution. The removal percentage decreased with an increase in the metal ion and steroid hormone concentration of the solution. This could be due to the availability of enough active sites on the surface of BASA550 at a lower concentration which enhances higher adsorption of the metal ions on the surface of the BASA550, while at higher metal ion concentration, the active surface sites of BASA550 become limited and thereby inhibiting the adsorption of the metal ions and steroid hormones resulting in lower removal percentage. However, with an increase in the metal ion and steroid hormone concentration of the solution, the number of metal ions and steroid hormones adsorbed per unit mass of the adsorbent increased. The ratio of the initial number of moles of metal ions and steroid hormones to the accessible surface area of the adsorbent is significant for low metal ion and steroid hormone concentrations, and fractional adsorption becomes independent of the initial concentration. Overall, the maximum percentage removal for Ni, Cd, and Pb achieved at a concentration of 0.5 mg/L was 92%, 85%, and 90%, respectively, while the highest adsorption capacity expressed in unit mass obtained for Ni, Cd, and Pb was 65 mg/g, 60 mg/g, and 65 mg/g, respectively, at 4.0 mg/L as shown in Fig. 10d. Similarly, the maximum percentage removal for estriol, α-estradiol, β-estradiol, testosterone, progesterone, and Bisphenol A achieved at a concentration of 0.5 mg/L was 87%, 85%, 85%, 87%, 81% and 85%, respectively, while the highest adsorption capacity expressed in unit mass obtained for estriol, α-estradiol, β-estradiol, testosterone, progesterone, and Bisphenol A was 62 mg/g, 62 mg/g, 57 mg/g, 59 mg/g, 58 mg/g, and 60 mg/g, respectively, at 4.0 mg/L as shown in Fig. 11d.

The effect of temperature on the adsorption is an important parameter to establish whether the adsorption of Ni, Cd, and Pb, and estriol, α-estradiol, β-estradiol, testosterone, progesterone, and Bisphenol A by BASA550 involves a chemical or only physical process. In this study, the effect of temperature was studied between 288 and 318 K with the aid of an ice bath and heating thermometric shaker. From the results shown in Fig. 10e, it can be observed that there was an increase in the adsorption capacity of BASA550 as the temperature increased until the optimum peak at 298 K with adsorption capacities of 42 mg/g, 40.5 mg/g, and 40 mg/g for Ni, Cd, and Pb, respectively and 40 mg/g, 39 mg/g, 38 mg/g, 39 mg/g, 37 mg/g, and 38.5 mg/g for estriol, α-estradiol, β-estradiol, testosterone, progesterone, and Bisphenol A as shown in Fig. 11e. This result showed that the increase in temperature could lead to bond breakage thereby increasing the active surface sites for adsorption of the heavy metal and steroid hormone, and thus increasing the adsorption capacities as the temperature increased [60].

The study of the effects of pH is crucial in determining the adsorption capacity in acidic, neutral, and alkaline media. In this study, the effect of pH on the adsorption capacity of the BASA500 was carried out in the pH range of 2–12. Figure 10f shows that the lowest adsorption capacity of BASA550 was observed in the acidic pH range; this could be due to the localised hydrogen ions (H^+^) which could make the surface of the BASA550 also positively charged. The heavy metal ions (Ni^2+^, Cd^2+,^ and Pb^2+^) were also positively charged; this could cause like charge repulsion effect and limit/inhibit the adsorption of the metal ions onto the surface of the BASA550. As the pH increased gradually from pH 2–8, the adsorption capacities also increased with the peak at pH (8); this finding was attributed to the introduction of hydroxyl ions (OH^−^) which started dominating the solution which could in turn make the surface of BASA550 negatively charged. The negatively charged surface of BASA550 would attract the metal ions with positive charges onto the available pores of the BASA550. After pH > 8, the adsorption capacities dropped mildly; this could be the result of mild precipitation of heavy metals in a strongly alkaline pH range. Figure 10f shows that the lowest adsorption capacity of BASA550 was observed in the basic pH range. It was found that the sorption capacity of steroid hormones was found at pH 2–6, which decreased at pH 8 and further decreased at pH 10–12. The adsorption capacity of steroid hormones by BASA550 decreased as the pH of the solution increased. This could be attributed to possible changes in the surface charges of BASA550 as well as the changes in the speciation of steroid hormones at various pH valuesas shown in Fig. 11f.

### Adsorption isotherm studies

Langmuir and Freundlich adsorption isotherms were used to study the adsorption data to properly evaluate and explain the sorbate-sorbent interaction at the interface [61]. To identify the best-fitting isotherm to examine the adsorption process, adsorption parameters were interpolated and statistically evaluated by using linear versions of the equation.

The Langmuir equation may be expressed in linear form as follows (Eq. 3):

3 $$\frac{{C}_{e}}{{\text{q}}_{\text{e}}}=\frac{1}{{\text{q}}_{\max}{\text{K}}_{\text{L}}}+ \frac{{\text{C}}_{\text{e}}}{{\text{q}}_{\max}}$$

where *q*_max_ (mg/g) denotes the maximum adsorption capacity, ${K}_{\text{L}}$ (L/mg) denotes the Langmuir constant, *q*_e_ is the amount of pollutants adsorbed at equilibrium (mg/g), and *C*_e_ is the equilibrium concentration of the adsorbate (mol/L).

A similar correlation was seen when $\frac{{\text{C}}_{\text{e}}}{{q}_{e}}$ was plotted against ${C}_{\text{e}}$, observed in Fig. 10a–c. The separation factor (${\text{R}}_{\text{L}}$) is a dimensionless constant that expresses the important attributes of the Langmuir isotherm given in Eq. (4) [62].

4 $${{\text{R}}_{\text{L}}} = \frac{1}{{1 + {{\text{K}}_{\text{L}}}{{\text{C}}_{\text{O}}}}}$$

where *C*_o_ is the initial solute concentration, *K*_L_ is the Langmuir constant, and ${R}_{\text{L}}$ is the separation factor. If ${R}_{\text{L}}$ = 0 the adsorption is considered irreversible, and the adsorption process is considered favourable when 0 < ${R}_{\text{L}}$ < 1, the adsorption is regarded as linear when ${R}_{\text{L}}$ = 1 and regarded as unfavourable if ${R}_{\text{L}}$ is higher than 1. In this study, the values of ${R}_{\text{L}}$ for all the heavy metals and steroid hormones are (0.0523–0.2074); 0 < ${R}_{\text{L}}$ < 1, indicating a favourable Langmuir adsorption process. The Langmuir and Freundlich adsorption isotherm parameters provided a good fit for the adsorption data. It was shown that the coefficient of correlation (R^2^) values of the two isotherm models Langmuir (R^2^ = 0.9291–0.9992) and Freundlich (R^2 ^= 0.9077–0.9974) were high and almost the same (Table 2). The high degree of agreement between the results and the adsorption models suggests that both Freundlich and Langmuir isotherm models fit the adsorption data very well. However, from the correlation coefficient values (R^2^) obtained, the Freundlich isotherm shows a higher correlation coefficient value for heavy metals, but the Langmuir isotherm shows a higher correlation coefficient for steroid hormones (Table 2).

The maximum adsorption capacity (*q*_max_ (mg/g)) obtained from the Langmuir isotherm was 35.21 mg/g, 41.84 mg/g, and 31.85 mg/g. for Cd, Ni, and Pb, respectively, and for the Freundlich isotherm, maximum adsorption capacity (*K*_F_ (mg/g)) of 35.80 mg/g, 38.19 mg/g, and 29.70 mg g^−1^ was obtained for Ni, Cd, and Pb, respectively. The maximum adsorption capacity obtained from the Langmuir isotherm was 83.19 mg/g, 79.30 mg/g, 63.65 mg/g, 77.70 mg/g, 84.39 mg/g, and 78.25 mg/g. for estriol, α-estradiol, β-estradiol, testosterone, progesterone, and Bisphenol A, respectively, For the Freundlich isotherm, a maximum adsorption capacity of 30.48 mg/g, 29.70 mg/g, 28.75 mg/g, 25.64 mg/g, 26.64 mg/g, and 29.70 mg/g. for estriol, α-estradiol, β-estradiol, testosterone, progesterone, and Bisphenol A, respectively.

The Freundlich isotherm may be used to describe adsorption processes on heterogeneous surfaces [62]. The surface heterogeneity and the exponential distribution of active sites and their energy are defined by this isotherm [63]. The Freundlich equation may be expressed in linear form as follows (Eq. 5):

5 $${\log}\left({\text{q}}_{\text{e}}\right)=\log{\text{K}}_{\text{f}}+ \frac{1}{\text{n}}{\log}\left({\text{C}}_{\text{e}}\right)$$

where *C*_e_ is the concentration of pollutants at equilibrium (mol/L), *q*_e_ represents the amount of pollutants absorbed at equilibrium (mg/g), *K*_F_ is the Freundlich constant and represents the adsorption capacity of the adsorbent, and 1/*n* represents the adsorption intensity of the adsorbent. A similar correlation was seen when $\text{log}\left({\text{q}}_{\text{e}}\right)$ was plotted against $\text{log}\left({\text{C}}_{\text{e}}\right)$ as revealed in Additional file 1: Figs. S1 and S2. The values of K_f_ > 1 for the Freundlich isotherm model suggest that adsorption was well favored during the removal of the steroid hormones and heavy metals. Additionally, the Freundlich linearity constants (1/*n*) for heavy metals and steroid hormones were all below one, suggesting that the adsorption of the pollutants on the ball-milled biochar is favourable.

**Table 2:** Langmuir and Freundlich adsorption isotherm values

Heavy metals	*q*_max_ (mg/g)	Langmuir				Freundlich	
		*K*_L_ (L/mg)	R_L_	R^2^	*K*_F_ (mg^−1^)	1/n	R^2^
Nickel	35.21	4.89	0.0927	0.9992	35.80	0.5712	0.9916
Cadmium	41.84	1.91	0.2074	0.9871	38.19	0.7610	0.9974
Lead	31.85	4.24	0.1054	0.9928	29.70	0.5501	0.9884
Estriol	83.19	3.29	0.1319	0.9844	30.48	0.5859	0.9826
α-Estradiol	79.30	7.16	0.0653	0.9494	29.70	0.5500	0.9856
β-Estradiol	63.65	5.07	0.0897	0.9931	28.75	0.5063	0.9819
Testosterone	77.70	2.41	0.1718	0.9291	25.64	0.5263	0.9077
Progesterone	84.39	2.21	0.1845	0.9476	26.64	0.5198	0.9158
Bisphenol A	78.25	9.06	0.0523	0.9645	29.70	0.5507	0.9855

### Adsorption kinetic studies

Adsorption kinetics describes the rate of adsorbate uptake on the adsorbent and assists in modelling the adsorption process. Chemical kinetics, also known as reaction kinetics, assesses the experimental factors that influence the overall speed of a chemical reaction and so, therefore, it assists in establishing equilibrium at a given time [64]. In this present study, pseudo-first-order and pseudo-second-order kinetics models were used to analyse experimental data as given in Eqs. (6) and (7).

The pseudo-first-order kinetic model is expressed as follows:

6 $${\log}\left({\text{q}}_{\text{e}}-{\text{q}}_{\text{t}}\right) =\log{\text{q}}_{\text{e}}- \frac{{\text{K}}_{1}}{2.303} \text{t}$$

where *q*_e_ and *q*_t_ are the amounts of adsorbate adsorbed (mg/g) at equilibrium, and at time t, respectively, and *K*_1_ is the first-order rate constant.

**Fig. 12:**
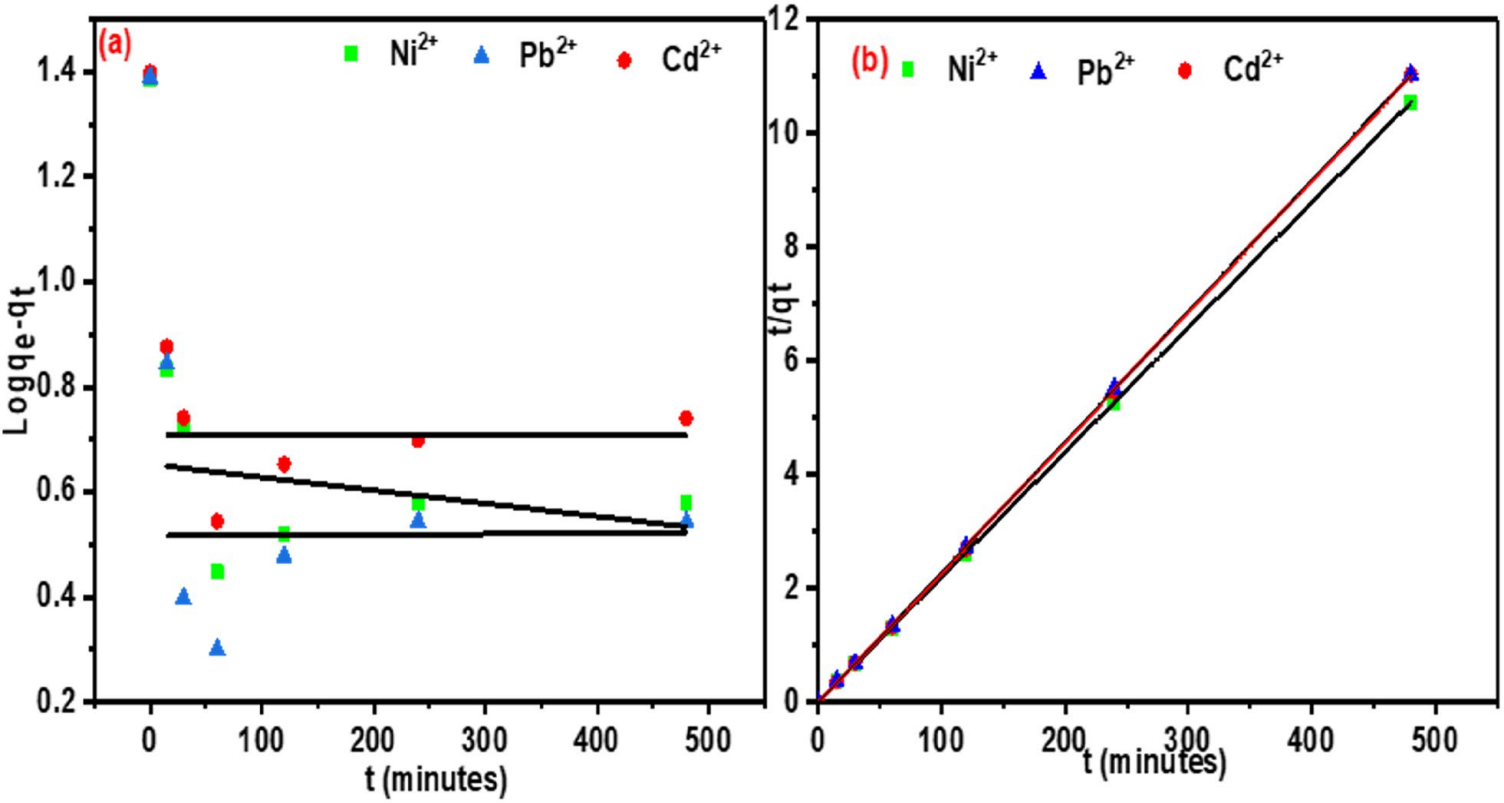
**a** Pseudo-first-order kinetics, and **b** pseudo-second-order kinetics

The pseudo-second-order kinetic model is expressed as follows:

7 $$\frac{\text{t}}{{\text{q}}_{\text{t}}} = \frac{1}{{\text{K}}_{2}{\text{q}}_{\text{e}}^{2}} + \frac{1}{{\text{q}}_{\text{e}}}\text{t}$$

where *q*_e_ and *q*_t_ are the amounts of heavy metals and steroid hormones adsorbed at equilibrium (mg/g) and at time *t* (min), respectively, and *K*_2_ is the second-order rate constant. The analysis of the data using pseudo-first-order and pseudo-second-order kinetic models brought about a broader understanding of the impact of time on the adsorption process. Based on the analysis of the experimental adsorption data using pseudo-first-order Fig. 12a and pseudo-second-order kinetic models, the pseudo-second-order kinetic model assumes that the rate-limiting step is chemical sorption or chemisorption. From the calculated results obtained, the pseudo-second-order model gave a straight-line curve as seen in Fig. 12b.

When comparing the pseudo-first-order and the pseudo-second-order model, the pseudo-second-order model accurately described the adsorption process using the coefficient of determination (R^2^) value of the pseudo-first-order model (R^2^ = 0.54297–0.9093) and the pseudo-second-order model (R^2^ = 0.9994–0.9999) as given in Table 3, which assume that chemisorption is the rate-determining step.

**Table 3:** Kinetic parameter values

Metal	Pseudo-first-order			Pseudo-second-order		
	*q*_e_ (mg/g)	K_1_	R^2^	*q*_e_	K_2_	R^2^
Nickel	6.79	0.001842	0.6485	45.45	0.0142	0.9997
Cadmium	7.54	0.001152	0.7538	43.48	0.0242	0.9999
Lead	5.37	0.001383	0.5429	43.48	0.0509	0.9996
Estriol	1.25	0.002263	0.8908	43.57	0.0098	0.9998
α-Estradiol	1.24	0.002042	0.8839	43.96	0.0107	0.9998
β- Estradiol	1.27	0.001974	0.8486	43.59	0.0076	0.9994
Testosterone	1.21	0.002464	0.9093	42.81	0.0121	0.9997
Progesterone	1.20	0.002372	0.8672	43.10	0.0309	0.9999
Bisphenol A	1.23	0.00194	0.8689	43.55	0.0064	0.9995

### Application of BASA550 for removal of cd, Ni, and Pb from a wastewater sample

The adsorption efficiency of the modified biochar was evaluated on wastewater samples to confirm its applicability in the treatment of heavy metal-contaminated wastewater samples by spiking 25 mL of wastewater sample (effluent and influent) with 2.5 mg/L standardised mixed solutions of three heavy metals (cadmium, nickel, and lead) and preparing a solution of these mixed heavy metals in deionised water. The percentage removal of heavy metals in deionised water, influent, and effluent using the BASA550 is shown in Table 4. For the solution of mixed metals prepared with deionised water, the removal percentage for cadmium, nickel, and lead, respectively, was recorded as 92.96%, 90.89%, and 90.29%, while the percentage removal in the influent was found to be 85.06%, 83.87%, and 84.73%, and 89.37%, 86.48%, and 87.40% in the effluent. The presence of numerous competing ions in the wastewater sample, which can likely be contending for the accessible active sites on the surface of the adsorbent, might be the cause of the slightly higher removal achieved from the standard mixed solution containing known amounts of heavy metals using deionised water. The result obtained was consistent with our previously reported findings of the application of ball-milled iron-based MOF/biochar composites for the removal of heavy metals in wastewater [65].

**Table 4:** Percentage removal of heavy metals in deionised water, real wastewater influent, and effluent using BASA550

Heavy metals	Deionised water (% removal)	Effluent (% removal)	Influent (% removal)
Cadmium	92.96	89.37	85.06
Nickel	90.89	86.48	83.87
Lead	90.22	87.40	84.73

### Application of BASA550 for removal of mixed steroid hormones in wastewater

The application of the ball-milled biochar was put to the test towards the removal of steroid hormones from real wastewater samples obtained from the wastewater treatment plant. The results obtained as shown in the Table 5 demonstrated that the adsorbent was shown to be effective in removing steroid hormones from the wastewater samples. The maximum percentage removal was recorded for the ultrapure water, ranging from 84.20 to 89.63%, while the removal percentages of between 78.91 and 87.81% and 73.58–84.51% were obtained for the spiked effluent and influent, respectively.

**Table 5:** Application of BASA550 for removal of mixed steroid hormones in effluent, influent and ultrapure water

Analytes	Ultrapure water (% removal)	Effluent (% removal)	Influent (% removal)
Estriol	88.38	87.81	84.51
α-Estradiol	85.51	81.37	75.28
β-Estradiol	89.63	83.46	73.58
Testosterone	84.20	78.91	76.34
Progesterone	88.73	85.47	78.71
Bisphenol A	87.14	82.66	80.38

### Reusability of the BASA550

**Fig. 13:**
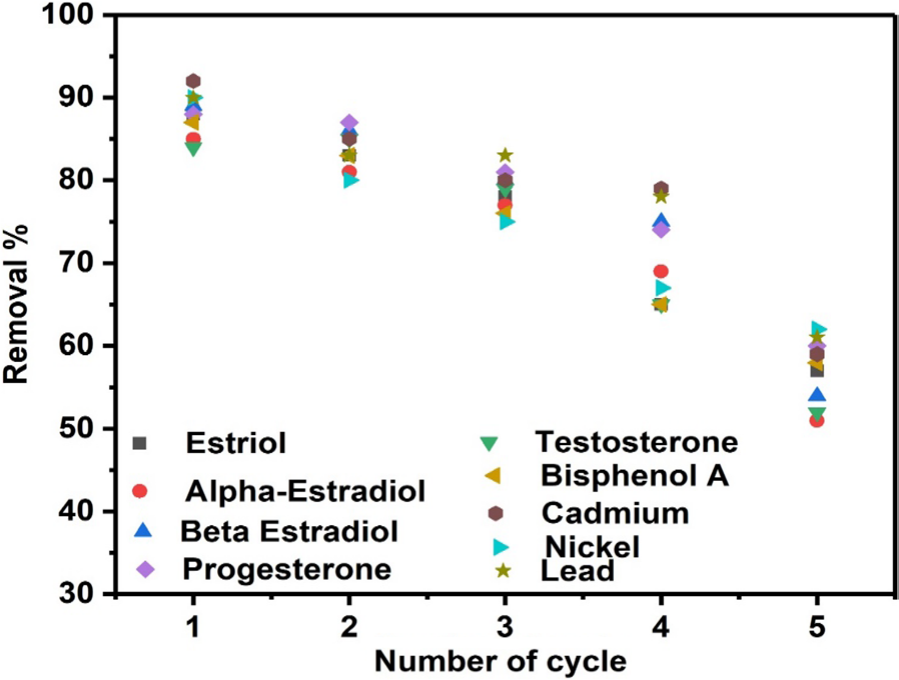
Reusability of BASA550 for the removal of steroid hormones and heavy metals regenerated by ethanol

High adsorption capacity and sustainable usage of an adsorbent are unique characteristics that a promising adsorbent should possess. The reusability of adsorbents is essential for a cost-effective method for long-term pollutant removal. Figure 13 depicts the adsorption performance of the regenerated BASA550. The adsorption capacity of regenerated biochar declined steadily, as can be seen in Fig. 13. When comparing the first and fourth cycles, the adsorption capacity of BASA550 only dropped significantly below 60% after the 4th cycle. The decrease in the adsorption capacity of the BASA550 after each desorption experiment can be ascribed to the loss of functional groups on the surface of the BASA550. The result of these experimental findings showed that the adsorbent (BASA550) could be successfully reused four times. As a result, the ball-milled modified biochar might be employed as a sustainable adsorbent for the simultaneous removal of steroid hormones and heavy metals in water and wastewater. Table 6 shows the comparison between the reported work and past studies using biochar for the removal of mixed heavy metals and steroid hormones.

### Proposed mechanism of adsorption

The adsorption mechanism of heavy metals from an aqueous solution using biochar is dependent on both the physical and chemical properties of the adsorbent. The physical adsorption achieved through the growth of micropores in the pore structure and high surface area of the adsorbent and the interaction between the adsorbent and heavy metals (adsorbate) is weak Vander wall force of attraction [66]. The chemical adsorption depends on the type of redox reaction that takes place between the adsorbent and the metal. The adsorption rate of biochar depends on the functional groups such as carboxyl, carbonyl, and hydroxide attached to the link. The disassociation of oxygen-containing functional groups, most of the time biochar carries a negative charge on their valency shell, which can comfortably remove the toxic heavy metals such as cadmium (Cd), lead (Pb), and nickel (Ni). The possible removal mechanism of steroid hormones could be attributed to the aromatic groups such as C=O and C=C at 1580 and 1620 cm^−1^ and C–H at 875 cm^−1^. The highly aromatic nature of biochar shows the π–π-electron donor and acceptor properties. The steroid hormones might fuse aromatic rings that are rich in π-electrons, and, consequently, the presence of π–π interactions between steroid hormones and biochar could be speculated. While H-bonding is formed between the phenolic and hydroxyl groups of steroid hormones and the hydroxyl, carbonyl, and carboxyl groups of the biochar surface. Therefore, π–π interactions and H–bonding may be the main adsorption mechanism of steroid hormones onto biochar.

**Table 6:** Comparison between the reported study and present studies using biochar for the removal of mixed heavy metals and steroid hormones

Biochar used	Contaminant	Percentage removal (%)	References
Peanut shell	Pb	95.96	[67]
Tamarind fruit shell	Pb	83.50	[68]
Sludge waste	Pb	57	[69]
Sludge waste	Cd	43	[69]
Corn cobs	Cd	85	[70]
Wheat straw	Cd	99	[71]
Sawdust biochar	Ni	83	[72]
Palm seed	Ni	87	[73]
Sugarcane straw, rice husk	Ni	24–72	[74]
Sludge waste	BPA	~ 80	[75]
Belles seed	Cd, Ni, and Pb	94–95	[25]
African star apple shell	Pb	84–90	Present study
African star apple shell	Cd	85–92	Present study
African star apple shell	Ni	83–90	Present study
African star apple shell	BPA	80–87	Present study
African star apple shell	Estriol	84–88	Present study
African star apple shell	Progesterone	78–88	Present study
African star apple shell	Testosterone	76–84	Present study
African star apple shell	Alpha estradiol	75–85	Present study
African star apple shell	Beta estradiol	73–89	Present study

## Conclusion

The various results obtained from characterisation analysis showed that the modified biochar contains C=O, C–O, and C–C which are the predominant functional groups in the derived biochar. The BET results showed that the surface area of the ball-milled biochar increased after modification, from 172 m^2^/g for the ASA550 to 302 m^2^/g for the BASA550, and consequently, enhanced the adsorption capacities of the ball-milled biochar. The Langmuir and Freundlich adsorption isotherms best described the experimental adsorption data with R_L_<1 and 1/*n* < 1 and a high degree of agreement of R^2^ data; Langmuir (R^2^ = 0.9291–0.9992) and Freundlich (R^2^ = 0.9077–0.9974).The adsorption kinetic studies using pseudo-first-order and pseudo-second-order models revealed that the pseudo-second-order model accurately described the adsorption process. The application of the BASA550 for the treatment of wastewater samples showed the highest removal percentages of 87.40%, 84.73%, and 92.96% for mixed heavy metals in effluent, influent, and DI water, respectively. Additionally, the results obtained for the treatment of wastewater containing steroid hormones indicated that the BASA550 showed the highest steroid hormone removal percentages of 87.81%, 84.51%, and 89.63% for effluent, influent, and DI water, respectively. The adsorbent (BASA550) had a reusability for the first four cycles. Consequently, ball-milled biochar from waste pods of African Star Apple (BASA550) could be applied for the removal of heavy metals and steroid hormones from wastewater.

### Supplementary Information

**Additional file 1: Figure S1.** Langmuir adsorption isotherm plot for (a) Ni, (b) Cd, and (c) Pb, and Freundlich adsorption isotherm plot for (d) Ni, (e) Cd, and (f) Pb. **Figure S2.** Freundlich adsorption isotherm for (a) Estriol, (b) α-Estradiol, (C) β-Estradiol, (d) Testosterone, (e) Progesterone (f) Bisphenol A hormones by ball milled biochar.**Figure S3.** Langmuir adsorption isotherm for (a) Estriol, (b) α-Estradiol, (C) β-Estradiol, (d) Testosterone, (e) Progesterone (f) Bisphenol A hormones by ball milled biochar. **Figure S4.** Pseudo-first-order kinetics for (a) Estriol, (b) α-Estradiol, (C) β-Estradiol, (d) Testosterone, (e) Progesterone (f) Bisphenol A hormones by ball milled biochar. **Figure S5.** Pseudo-second-order kinetics for (a) Estriol, (b) α-Estradiol, (C) β-Estradiol, (d) Testosterone, (e) Progesterone (f) Bisphenol A hormones by ball milled biochar.

## Data Availability

Data will be provided upon request.
