# Supplementary material for: Facile solvent-free modified biochar for removal of mixed steroid hormones and heavy metals: isotherm and kinetic studies
Source: BMC Chem. 2023 Nov 20;17(1):158. doi: 10.1186/s13065-023-01071-5 (PMC10662544; doi:10.1186/s13065-023-01071-5)
Supplement: Supplementary file 1 — Additional file 1: Figure S1. Langmuir adsorption isotherm plot for (a) Ni, (b) Cd, and (c) Pb, and Freundlich adsorption isotherm plot for (d) Ni, (e) Cd, and (f) Pb. Figure S2. Freundlich adsorption isotherm for (a) Estriol, (b) α-Estradiol, (C) β-Estradiol, (d) Testosterone, (e) Progesterone (f) Bisphenol A hormones by ball milled biochar.Figure S3. Langmuir adsorption isotherm for (a) Estriol, (b) α-Estradiol, (C) β-Estradiol, (d) Testosterone, (e) Progesterone (f) Bisphenol A hormones by ball milled biochar. Figure S4. Pseudo-first-order kinetics for (a) Estriol, (b) α-Estradiol, (C) β-Estradiol, (d) Testosterone, (e) Progesterone (f) Bisphenol A hormones by ball milled biochar. Figure S5. Pseudo-second-order kinetics for (a) Estriol, (b) α-Estradiol, (C) β-Estradiol, (d) Testosterone, (e) Progesterone (f) Bisphenol A hormones by ball milled biochar. [file 13065_2023_1071_MOESM1_ESM.docx]

**Facile solvent-free ball-milled modified biochar for removal of mixed steroid hormones and heavy metals: Isotherm and kinetic studies.**

Sefiu Olaitan Amusat^1*^, Temesgen Girma Kebede^1^, Edward Ndumiso Nxumalo^2^, Simiso Dube^1^, and Mathew Muzi Nindi^2*^

^1^Department of Chemistry, College of Science, Engineering, and Technology, University of South Africa, The Science Campus, Florida Park, Corner Christian de Wet & Pioneer Avenue, Florida, 1709, South Africa

^2^Institute for Nanotechnology and Water Sustainability (iNanoWS), College of Science, Engineering, and Technology, The Science Campus, University of South Africa, Corner Christian de Wet & Pioneer Avenue Florida Park, 1709, South Africa

****Corresponding authors:*** [*amusatolaitan@gmail.com*](mailto:amusatolaitan@gmail.com)*; nindim@unisa.ac.za.*

**Additional file 1**


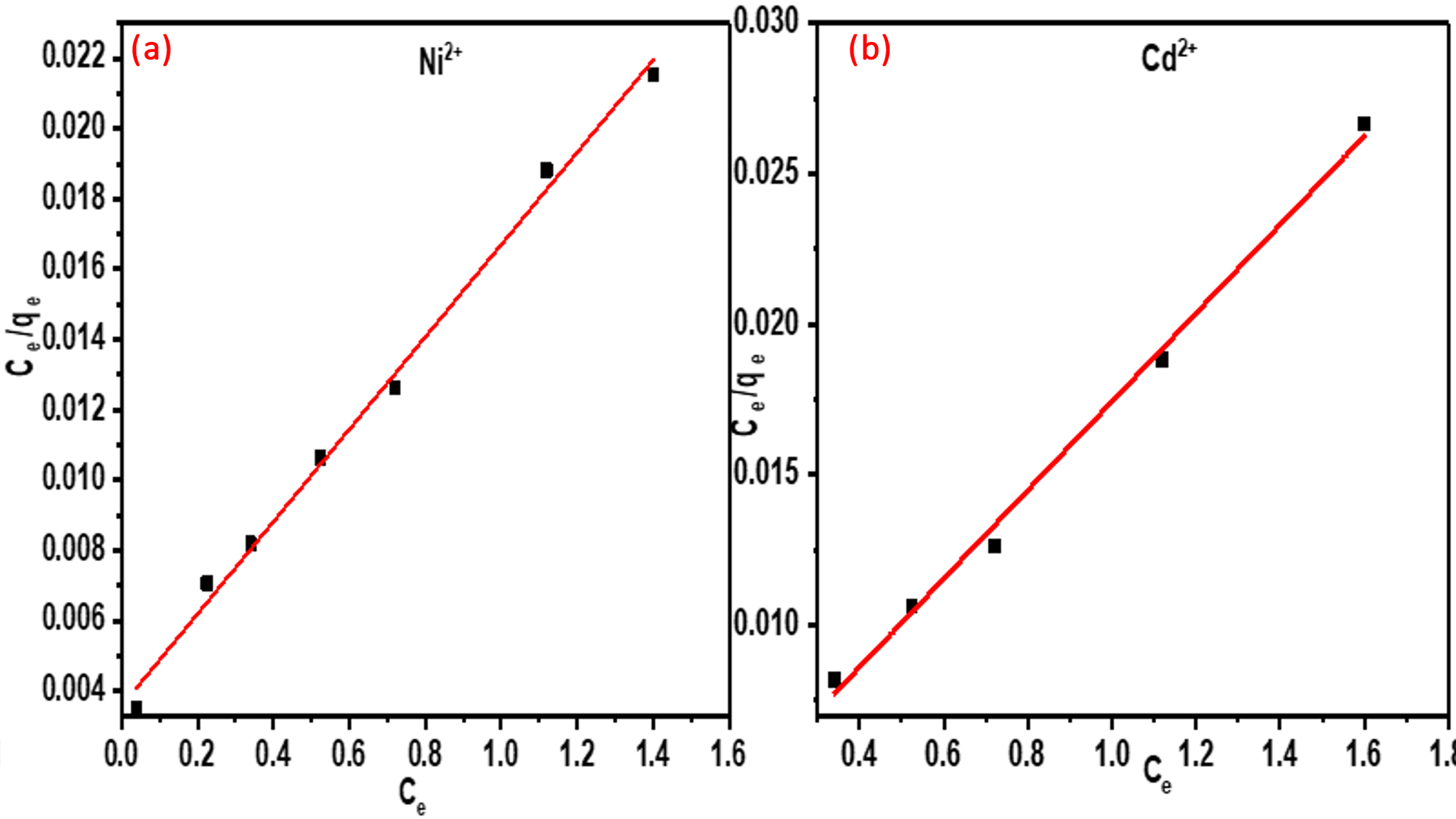


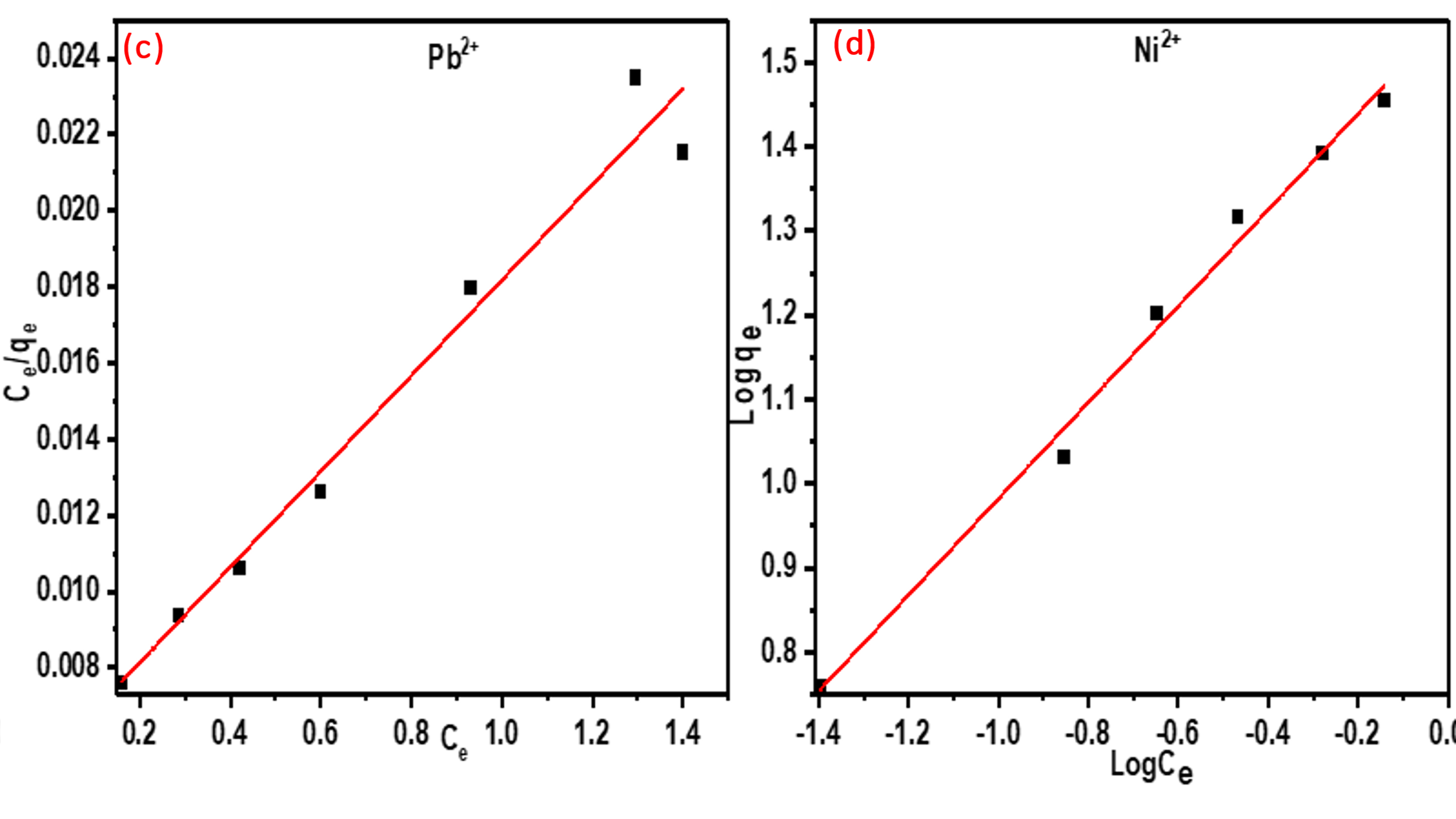

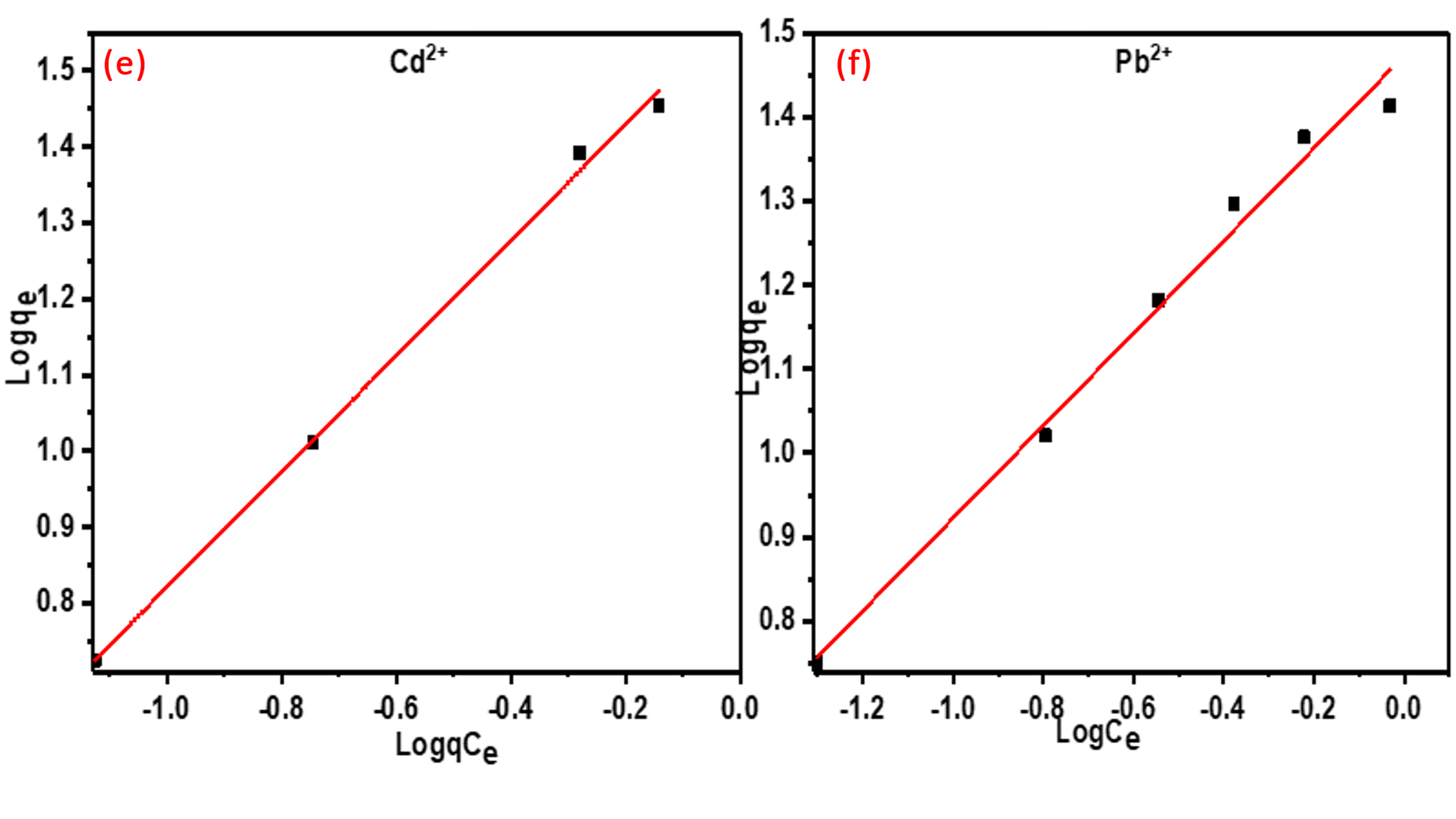


Figure S1: Langmuir adsorption isotherm plot for (a) Ni, (b) Cd, and (c) Pb, and Freundlich adsorption isotherm plot for (d) Ni, (e) Cd, and (f) Pb


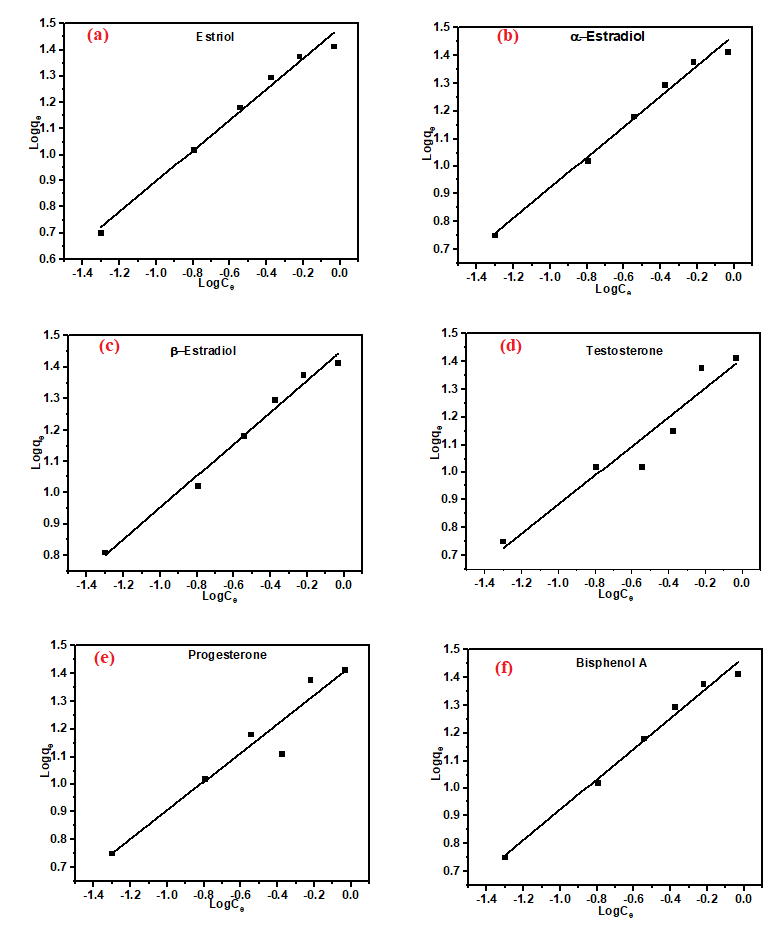


**Figure S2: Freundlich adsorption isotherm for (a) Estriol, (b) α-Estradiol, (C) β-Estradiol, (d) Testosterone, (e) Progesterone (f) Bisphenol A hormones by ball milled biochar.**

**
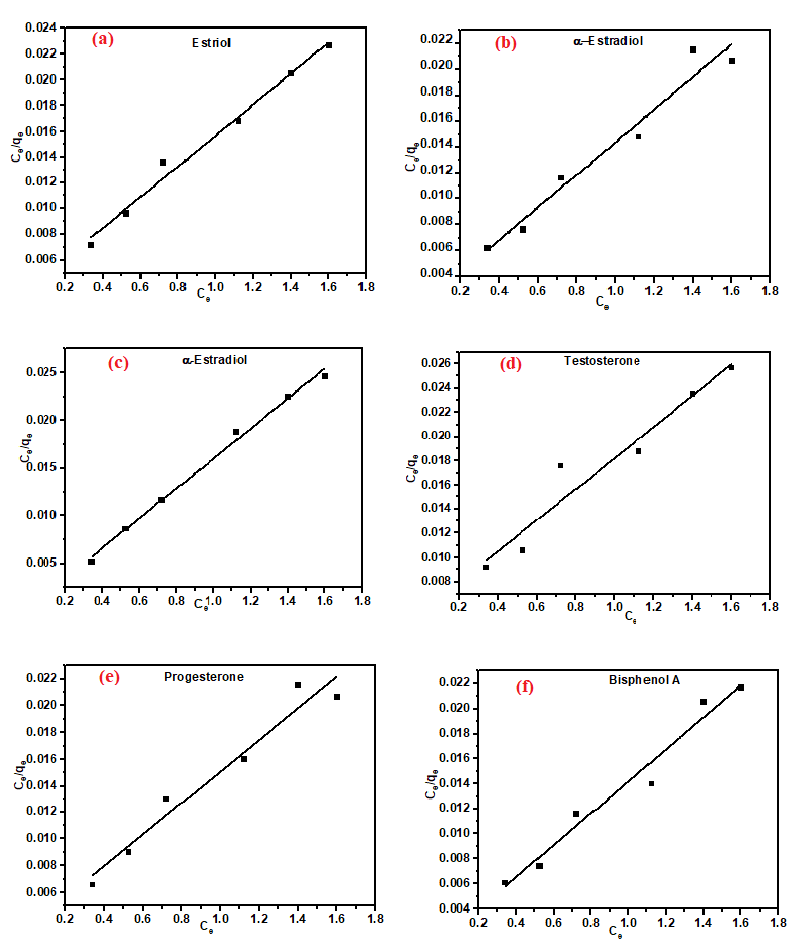
**

**Figure S3: Langmuir adsorption isotherm for (a) Estriol, (b) α-Estradiol, (C) β-Estradiol, (d) Testosterone, (e) Progesterone (f) Bisphenol A hormones by ball milled biochar.**

**
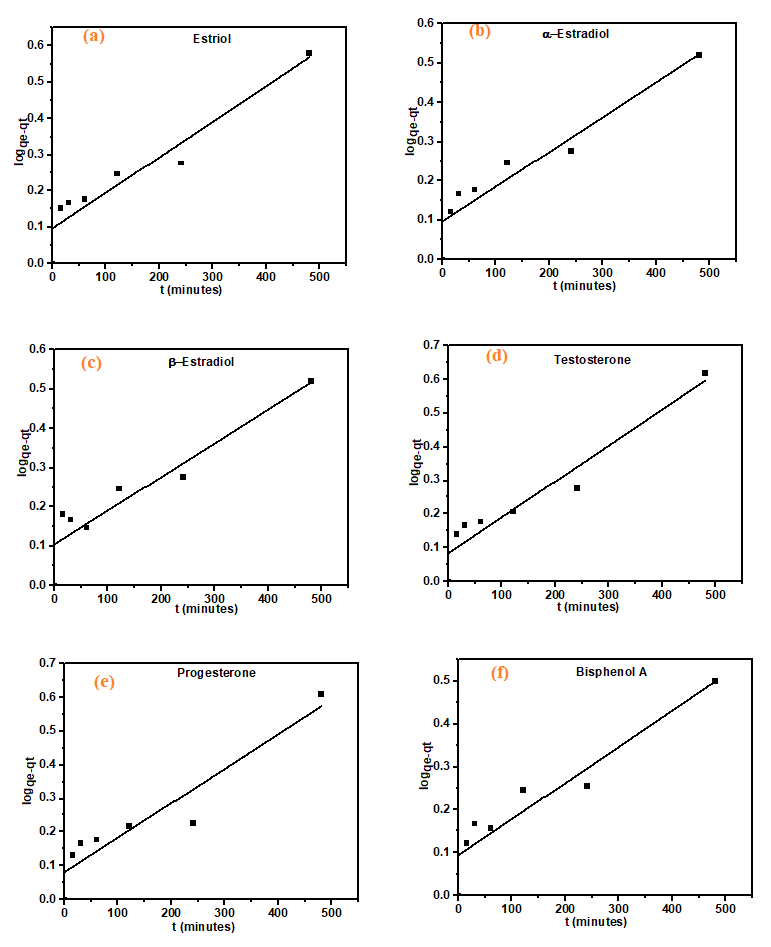
**

**Figure S4: Pseudo-first-order kinetics for (a) Estriol, (b) α-Estradiol, (C) β-Estradiol, (d) Testosterone, (e) Progesterone (f) Bisphenol A hormones by ball milled biochar.**

**
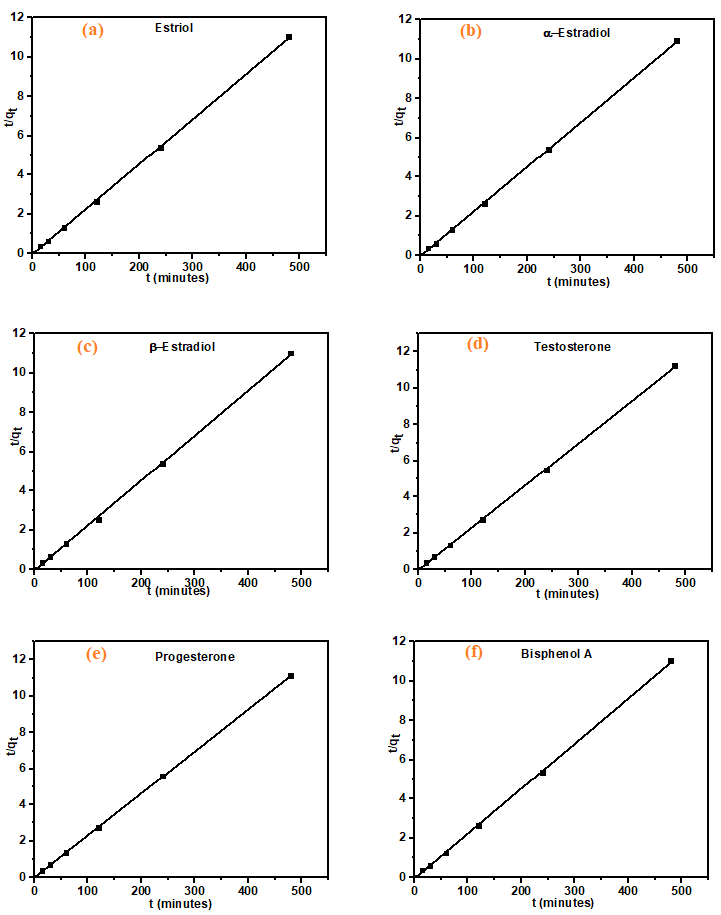
**

**Figure S5: Pseudo-second-order kinetics for (a) Estriol, (b) α-Estradiol, (C) β-Estradiol, (d) Testosterone, (e) Progesterone (f) Bisphenol A hormones by ball milled biochar.**
